# Mapping Lesion-Related Epilepsy to a Human Brain Network

**DOI:** 10.1001/jamaneurol.2023.1988

**Published:** 2023-07-03

**Authors:** Frederic L. W. V. J. Schaper, Janne Nordberg, Alexander L. Cohen, Christopher Lin, Joey Hsu, Andreas Horn, Michael A. Ferguson, Shan H. Siddiqi, William Drew, Louis Soussand, Anderson M. Winkler, Marta Simó, Jordi Bruna, Sylvain Rheims, Marc Guenot, Marco Bucci, Lauri Nummenmaa, Julie Staals, Albert J. Colon, Linda Ackermans, Ellen J. Bubrick, Jurriaan M. Peters, Ona Wu, Natalia S. Rost, Jordan Grafman, Hal Blumenfeld, Yasin Temel, Rob P. W. Rouhl, Juho Joutsa, Michael D. Fox

**Affiliations:** 1 Center for Brain Circuit Therapeutics, Departments of Neurology, Psychiatry and Radiology, Brigham and Women’s Hospital, Boston, Massachusetts; 2 Harvard Medical School, Harvard University, Boston, Massachusetts; 3 Department of Neurology and School for Mental Health and Neuroscience, Maastricht University Medical Center, Maastricht, the Netherlands; 4 Turku Brain and Mind Center, Department of Clinical Neurophysiology, Clinical Neurosciences, Turku University Hospital and University of Turku, Turku, Finland; 5 Department of Neurology, Boston Children’s Hospital, Boston, Massachusetts; 6 Computational Radiology Laboratory, Department of Radiology, Boston Children’s Hospital, Harvard Medical School, Boston, Massachusetts; 7 National Institute of Mental Health, National Institutes of Health, Bethesda, Maryland; 8 Department of Human Genetics, University of Texas Rio Grande Valley, Brownsville; 9 Neuro-Oncology Unit, Hospital Universitari de Bellvitge - Institut Català d’Oncologia (IDIBELL), L’Hospitalet del Llobregat, Barcelona, Spain; 10 Department of Functional Neurology and Epileptology, Lyon Neurosciences Research Center, Hospices Civils de Lyon and University of Lyon, Lyon, France; 11 Institut national de la santé et de la recherche médicale, Lyon, France; 12 Department of Functional Neurosurgery, Hospices Civils de Lyon and University of Lyon, Lyon, France; 13 Turku PET Centre, University of Turku and Åbo Akademi University, Turku, Finland; 14 Division of Clinical Geriatrics, Center for Alzheimer Research, Department of Neurobiology, Care Sciences and Society, Karolinska Institutet, Stockholm, Sweden; 15 Department of Psychology, University of Turku, Turku, Finland; 16 Academic Center for Epileptology Kempenhaeghe/Maastricht University Medical Center, Heeze & Maastricht, the Netherlands; 17 Department of Epileptology, Centre Hospitalier Universitaire Martinique, Fort-de-France, France; 18 Department of Neurosurgery and School for Mental Health and Neuroscience, Maastricht University Medical Center, Maastricht, the Netherlands; 19 Athinoula A Martinos Center for Biomedical Imaging, Department of Radiology, Massachusetts General Hospital, Charlestown, Massachusetts; 20 J. Philip Kistler Stroke Research Center, Department of Neurology, Massachusetts General Hospital, Boston, Massachusetts; 21 Cognitive Neuroscience Laboratory, Think + Speak Lab, Shirley Ryan Ability Lab, Chicago, Illinois; 22 Department of Physical Medicine and Rehabilitation, Feinberg School of Medicine, Northwestern University, Chicago, Illinois; 23 Departments of Neurology, Neuroscience and Neurosurgery, Yale School of Medicine, New Haven, Connecticut; 24 Berenson-Allen Center for Noninvasive Brain Stimulation, Department of Neurology, Beth Israel Deaconess Medical Center, Boston, Massachusetts

## Abstract

**Importance:**

It remains unclear why lesions in some locations cause epilepsy while others do not. Identifying the brain regions or networks associated with epilepsy by mapping these lesions could inform prognosis and guide interventions.

**Objective:**

To assess whether lesion locations associated with epilepsy map to specific brain regions and networks.

**Design, Setting, and Participants:**

This case-control study used lesion location and lesion network mapping to identify the brain regions and networks associated with epilepsy in a discovery data set of patients with poststroke epilepsy and control patients with stroke. Patients with stroke lesions and epilepsy (n = 76) or no epilepsy (n = 625) were included. Generalizability to other lesion types was assessed using 4 independent cohorts as validation data sets. The total numbers of patients across all datasets (both discovery and validation datasets) were 347 with epilepsy and 1126 without. Therapeutic relevance was assessed using deep brain stimulation sites that improve seizure control. Data were analyzed from September 2018 through December 2022. All shared patient data were analyzed and included; no patients were excluded.

**Main Outcomes and Measures:**

Epilepsy or no epilepsy.

**Results:**

Lesion locations from 76 patients with poststroke epilepsy (39 [51%] male; mean [SD] age, 61.0 [14.6] years; mean [SD] follow-up, 6.7 [2.0] years) and 625 control patients with stroke (366 [59%] male; mean [SD] age, 62.0 [14.1] years; follow-up range, 3-12 months) were included in the discovery data set. Lesions associated with epilepsy occurred in multiple heterogenous locations spanning different lobes and vascular territories. However, these same lesion locations were part of a specific brain network defined by functional connectivity to the basal ganglia and cerebellum. Findings were validated in 4 independent cohorts including 772 patients with brain lesions (271 [35%] with epilepsy; 515 [67%] male; median [IQR] age, 60 [50-70] years; follow-up range, 3-35 years). Lesion connectivity to this brain network was associated with increased risk of epilepsy after stroke (odds ratio [OR], 2.82; 95% CI, 2.02-4.10; *P* < .001) and across different lesion types (OR, 2.85; 95% CI, 2.23-3.69; *P* < .001). Deep brain stimulation site connectivity to this same network was associated with improved seizure control (*r*, 0.63; *P* < .001) in 30 patients with drug-resistant epilepsy (21 [70%] male; median [IQR] age, 39 [32-46] years; median [IQR] follow-up, 24 [16-30] months).

**Conclusions and Relevance:**

The findings in this study indicate that lesion-related epilepsy mapped to a human brain network, which could help identify patients at risk of epilepsy after a brain lesion and guide brain stimulation therapies.

## Key Points

**Question:**

Does lesion-related epilepsy map to a brain network?

**Findings:**

In this case-control study of lesion locations in patients who either developed epilepsy or did not, lesions associated with epilepsy occurred in multiple heterogenous brain locations. However, these same lesion locations were part of a specific brain network defined by functional connectivity to the basal ganglia and cerebellum, and deep brain stimulation sites associated with seizure control were connected to this same network.

**Meaning:**

The findings indicate that lesion-related epilepsy mapped to a brain network that could help identify patients at risk of epilepsy after a brain lesion and guide brain stimulation therapies.

## Teaser

This case-control study evaluates the locations of epilepsy-associated lesions with respect to specific brain regions and networks.

## Introduction

Focal epilepsy affects more than 30 million patients worldwide and is commonly caused by brain lesions, such as stroke.^1^ However, it is unclear why some lesion locations cause epilepsy while others do not.^2^

Identifying lesion locations at increased or decreased risk of epilepsy is important for 3 reasons. First, it may help refine models designed to predict epilepsy risk,^3^ allowing for better prognosis or early intervention. Second, it may lend mechanistic insight into why some lesion locations but not others lead to epilepsy.^2^ Third, brain lesions can help identify or refine therapeutic targets for brain stimulation,^4,5,6^ and played a role in identifying the thalamus as a therapeutic target for epilepsy.^7,8^ Given that brain stimulation outcomes in epilepsy remain heterogenous,^9^ mapping lesions that cause or do not cause epilepsy may help identify regions or networks that could be targeted for seizure control.

Lesion mapping methods have improved in recent years.^4,10^ Voxel-based lesion symptom mapping can test whether lesions causing a specific symptom intersect specific brain regions.^10^ Lesion network mapping can test whether lesions causing a specific symptom intersect specific brain networks and can thus detect associations that go beyond individual brain regions.^11^ This latter technique uses a wiring diagram of the human brain termed the *human connectome* to identify network connections common across different lesion locations. It has proven particularly useful when lesions in different locations cause a similar symptom and has identified effective therapeutic targets for brain stimulation.^6,12,13,14^ Here, we use these lesion-mapping techniques to assess whether lesion locations associated with epilepsy map to specific brain regions and networks.

## Methods

This multicenter study was carried out in accordance with the Declaration of Helsinki, approved by the institutional review board of the Brigham and Women’s Hospital, Boston, Massachusetts, and exempted from obtaining informed consent based on the secondary use of research data. Strengthening the Reporting of Observational Studies in Epidemiology (STROBE) reporting guidelines for case-control studies were followed. For full details on each data set and analysis, see the eMethods in Supplement 1.

### Patients With Stroke and Brain Lesions

**Figure 1.**
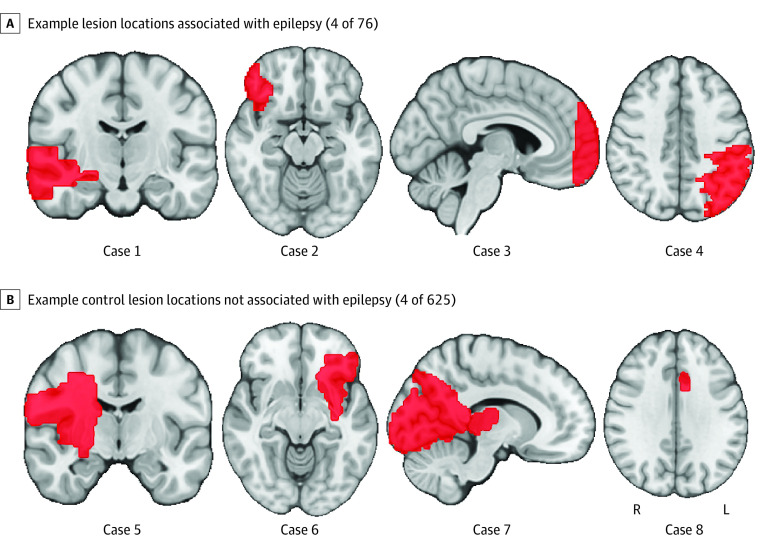
Lesion Locations Brain slices are shown in radiological orientation.

In this case-control study, we studied lesion locations from 76 patients with poststroke epilepsy (ischemia) who were part of a previous study^15^ (Figure 1A). To control for the normal distribution of stroke lesions, 2 independent and previously published cohorts of patients with consecutive stroke and lesion locations not associated with epilepsy were used as controls (n = 135,^16^ n = 490),^17^ as in our prior work^14,18^ (Figure 1B). Patient demographic characteristics of this discovery data set (n = 701) are presented in eTable 1 in Supplement 1.

### Lesion Location Mapping

Lesion location mapping methods were used to test whether lesions associated with poststroke epilepsy map to a specific brain region.^10^ We assessed lesion overlap (or damage) of each lesion to the cortex, subcortex, cortical lobes and vascular territories. Since larger lesions are more likely to lead to epilepsy,^19^ we controlled for lesion volume in all analyses. To identify any lesioned brain regions or voxels associated with epilepsy, we performed voxel-based lesion symptom mapping using both univariate and multivariate methods, correcting for lesion volume.^20,21,22^

### Lesion Network Mapping

**Figure 2.**
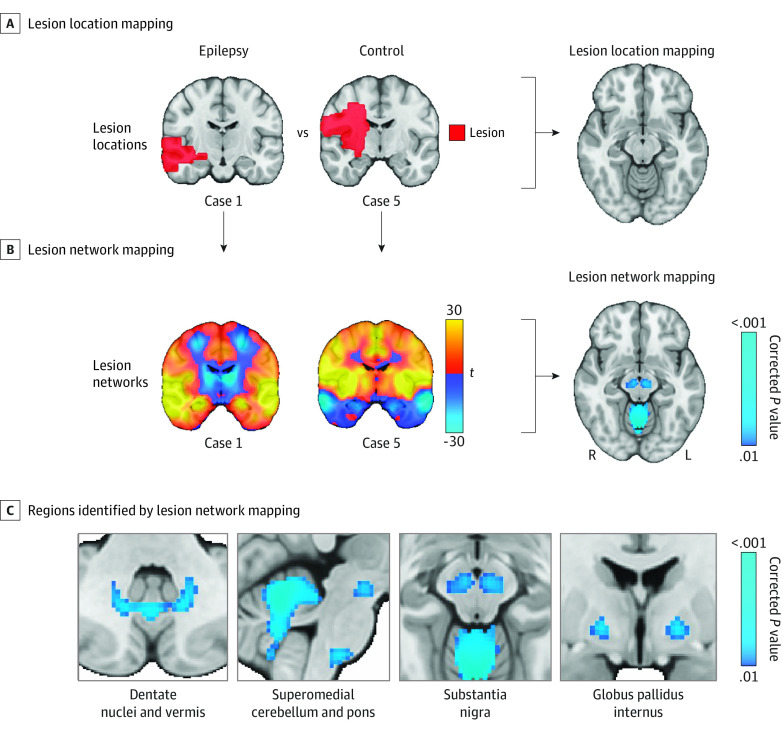
Lesion Location and Network Mapping A, Lesion location mapping methods were not able to identify associations between damage to a specific brain region and epilepsy. B, Lesion network mapping was then performed, which computes the functional connectivity between each lesion location (red) and all other brain voxels, using the resting-state functional connectivity data from 1000 healthy participants (ie, the human connectome). C, Lesion network mapping identified regions in the basal ganglia and cerebellum (ie, lesion network nodes) that were more negatively connected to lesion locations associated with epilepsy vs control lesions; 2-sided *P* values are shown after familywise error rate correction for multiple testing. Brain slices are shown in radiological orientation.

Lesion network mapping was used to test whether lesions associated with poststroke epilepsy map to a specific brain network. As described previously,^11,23,24^ we computed seed-based functional connectivity between each lesion location and all other brain voxels using the resting-state functional connectivity data (2 × 2 × 2-mm resolution) from 1000 healthy participants (human brain connectome from the Brain Genomics Superstruct Project: https://dataverse.harvard.edu/dataverse/GSP).^25,26^ This process results in a lesion network for each lesion location (Figure 2A and 2B; eFigure 1 in Supplement 1). To identify the functional connections (ie, lesion network nodes) associated with epilepsy, we performed a voxel-based permutation test on a whole-brain level using the software Permutation Analysis of Linear Models (PALM) (https://fsl.fmrib.ox.ac.uk/fsl/fslwiki/PALM)^27^ and controlled for lesion volume as a covariate, as in our prior work.^14,18^

For detailed description of the control analyses, see the eMethods in Supplement 1. We performed multiple control analyses to assess the consistency of our lesion network mapping findings using different control data sets, connectome preprocessing methods, covariates, and subgroups. We used statistical mediation analysis to determine the association between lesion connectivity, lesion volume, and damage to the cortex or subcortex. Finally, we repeated lesion network mapping using a structural connectome instead of a functional connectome and tested convergence.^28^

### Generalizability to Other Lesion Types

To test for generalizability of the lesion network nodes derived from ischemic stroke data (ie, the discovery data set) (Figure 2C) to other lesion types, we studied 4 previously published cohorts of other lesion etiologies associated with epilepsy: hematomas,^29^ traumas,^30^ tumors,^31^ and tubers^32^ (validation data sets totaling 772 participants, 271 [35%] with epilepsy). Patient demographic characteristics for these 4 validation data sets are presented in eTable 2 in Supplement 1.

**Figure 3.**
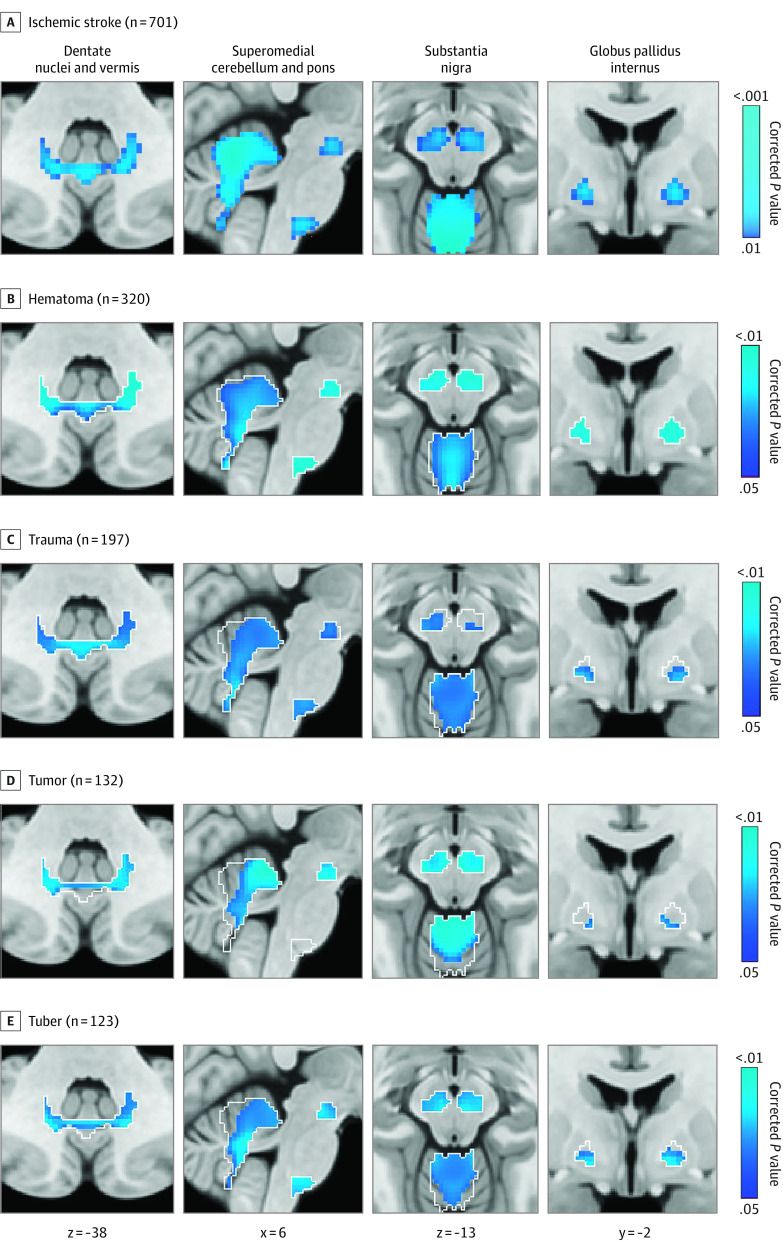
Generalizability to Other Lesion Types Lesion network nodes in the basal ganglia and cerebellum derived from ischemic stroke data (A) were used as an a priori search space (white outlines) to test for similar findings in four validation data sets with different lesion etiologies (B). Negative functional connectivity to voxels in the basal ganglia and cerebellum was significantly associated with epilepsy in hematomas, traumas, tumors, and tubers. One-sided *P* values are shown after false discovery rate correction for multiple testing. Brain slices are shown in radiological orientation.

Using the lesion network nodes derived from poststroke epilepsy as an a priori region of interest (Figure 2C), we tested the hypothesis that each of the other lesion types would show similar functional connections associated with epilepsy (Figure 3). This voxel-based PALM analysis was repeated on a whole-brain level after combining all 4 validation data sets, controlling for data set and lesion volume.

### Estimating Risk of Lesion-Related Epilepsy

**Figure 4.**
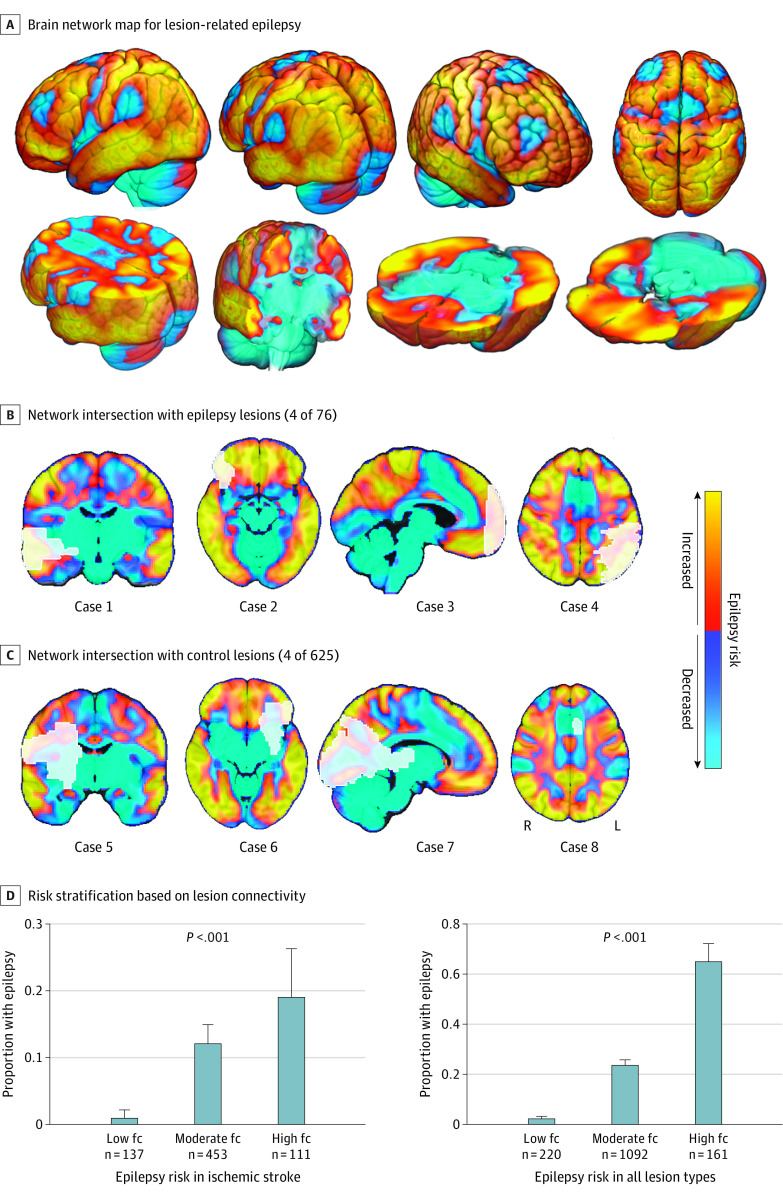
Relevance for Estimating Epilepsy Risk A, Functional connectivity (fc) with the lesion network nodes in the basal ganglia and cerebellum (Figure 2C) defines a distributed brain network map of areas at increased risk or decreased risk of epilepsy when lesioned. Regions of increased risk in this network include the temporal lobe, parietal lobe, areas around the central sulcus, and CA1 region of the hippocampus. Regions of decreased risk include the supplementary motor area, anterior cingulate, and subcortical regions. To illustrate this finding, we show the same lesion locations from Figure 1 (white outlines), now overlaid on our network map, including 5 representative lesions associated with epilepsy (B) and 5 lesions not associated with epilepsy (C). Note that the lesions associated with epilepsy intersect areas of high risk compared to lesions not associated with epilepsy. D, Patients were stratified into 3 risk groups based on intersection of their lesion location with this network, using leave-one–data set–out analysis. More patients in the high-fc group had epilepsy compared to patients in the low-fc group both for ischemic stroke and across all lesion types.

Functional connectivity of the lesion network nodes defines a distributed brain network map with regions of increased or decreased risk of epilepsy (Figure 4A-C). To evaluate the potential prognostic relevance of this network map, we calculated lesion connectivity values using a leave-one–data set–out analysis. For an expanded explanation of leave-one–data set–out analysis, see the eMethods in the Supplement 1. Lesion connectivity values were calculated by computing the functional connectivity between each lesion from a left-out data set to the lesion network nodes generated from the other 4 data sets (ie, region of interest–to–region of interest connectivity). This analysis tests whether lesion connectivity is associated with risk of epilepsy in an out-of-sample manner, and can be illustrated as intersection of lesion locations with our brain network map (Figure 4). Patients were then stratified into 3 categories of high, low, and moderate lesion connectivity (1 SD above or below the mean and in between, respectively) and the proportion of epilepsy was compared across categories, similar to previous work.^14^

### Therapeutic Relevance for Deep Brain Stimulation

**Figure 5.**
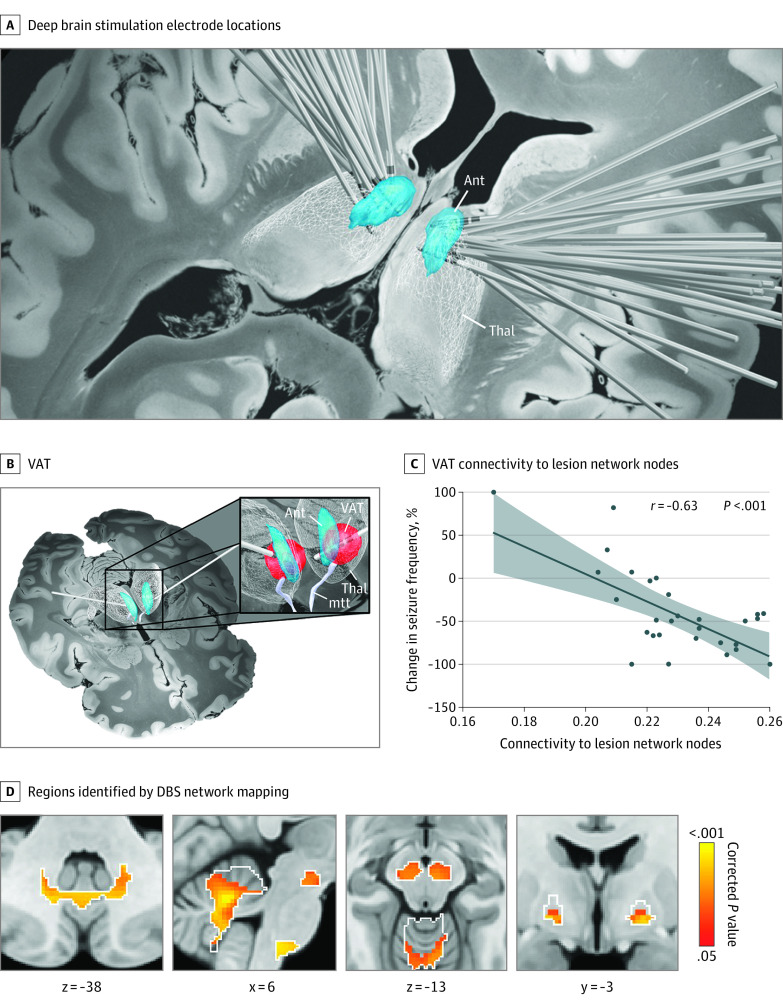
Relevance for Deep Brain Stimulation (DBS) in Epilepsy A, DBS electrodes from 30 patients with drug-resistant epilepsy show slight variability in electrode location within the anterior thalamus. B, The stimulation site for each patient was identified by computing the volume of activated tissue based on individualized stimulation settings. C, Functional connectivity between patient-specific stimulation sites and the lesion network nodes in the basal ganglia and cerebellum was associated with better seizure outcome. D, Positive functional connectivity between patient-specific stimulation sites and multiple voxels within the lesion network nodes (white outlines) was significantly associated with therapeutic response after deep brain stimulation. One-sided *P* values are shown after false discovery rate correction for multiple testing. Brain slices are shown in radiological orientation. Ant indicates anterior nucleus of the thalamus; mtt, mammillothalamic tract; Thal, thalamus; VAT, volume of activated tissue.

To evaluate the potential therapeutic relevance of this network, we analyzed a cohort of 30 patients who received anterior thalamic deep brain stimulation (DBS) for drug-resistant focal epilepsy.^33^ DBS electrode locations and stimulation sites were localized using Lead-DBS (https://www.lead-dbs.org), similar to prior work^34,35^ (Figure 5A and B). Patient demographics are presented in eTable 3 in Supplement 1. We computed the functional connectivity of the DBS sites (volume of tissue activated) to our lesion network nodes (Figure 2C) using region of interest–to–region of interest connectivity and tested for association with clinical outcome (percentage of change in seizure frequency) (Figure 5C). Next, we performed a voxel-based analysis using PALM to identify connections associated with improved seizure control (Figure 5D). This analysis was performed both within the a priori region of interest derived from lesion network mapping (Figure 2C) and using a whole-brain analysis.

### Statistical Analysis

Statistical analyses were performed in R version 2022.12 (R Foundation) and MATLAB version 2020b (MathWorks). Power analyses were performed in G*Power version 3.1 (Heinrich Heine Universität Düsseldorf).^36^ Data were analyzed from September 2018 through December 2022.

Group differences in lesion volume, damage to brain regions, or functional connectivity on a voxel-wise level were tested using an Aspin-Welch test, and the V statistic was reported. To assess the association between lesion connectivity and epilepsy, multivariate models were fitted with logistic regression and corrected for potential confounders as covariates. Statistical mediation analysis was performed to assess the relationship between epilepsy, lesion connectivity, and covariates. Proportions of patients with epilepsy across categories were compared using a χ^2^ test. To ensure results were independent of category cutoffs, we computed receiver operating characteristics. Model discrimination was calculated as the area under the curve. The association between DBS connectivity and clinical outcome was calculated using a Pearson correlation (*r*) and repeated excluding outliers. Two-sided *P* values less than .05 were considered significant, unless otherwise stated. Higher statistical thresholds were often used to highlight the most significant findings (Figure legends). Significance was assessed using permutations and correction for multiple testing.

## Results

### Lesion Location Mapping

Lesion locations associated with poststroke epilepsy were heterogeneously distributed across the brain (Figure 1) with a maximum lesion overlap of 24% (18 of 76) (eFigure 2 in Supplement 1). Control lesions were also heterogenous, with a maximum overlap of 16% (98 of 625). Lesions associated with epilepsy were larger than control lesions (V, 4.8; corrected *P* = .001) (eTable 4 in Supplement 1). After controlling for lesion volume, lesions associated with epilepsy damaged more of the cortex (V, 4.9; corrected *P* < .001) and less of the subcortex (V, −4.6; corrected *P* < .001), but there were no differences for a specific lobe (including mesial temporal lobe) or vascular territory (Figure 1; eFigures 3 and 4 in Supplement 1). Voxel-based lesion symptom mapping was not able to identify any lesioned brain regions or individual voxels statistically associated with epilepsy (Figure 2A).

### Lesion Network Mapping

Functional connectivity between lesion locations and regions in the basal ganglia and cerebellum was strongly associated with poststroke epilepsy (maximum V, 6.8; peak corrected *P* < .001) (Figure 2B and C). Specifically, lesion locations related to epilepsy were more negatively connected (ie, anticorrelated) to the substantia nigra, globus pallidus internus, and cerebellum (superomedial cerebellum, dentate nuclei, vermis) compared to control lesions. We refer to these regions as lesion network nodes.

Results were consistent across different control analyses (eFigures 5-8 in Supplement 1). Lesion connectivity fully mediated the association between epilepsy, lesion volume, and damage to the cortex or subcortex (eFigure 9 in Supplement 1). Voxel-based lesion symptom mapping results, using liberal statistical cutoffs, were consistent with lesion network mapping results but only identified part of the network (eFigure 10 in Supplement 1). Lesion network mapping using a structural connectome converged on a similar network (eFigure 11 in Supplement 1).

### Generalizability to Other Lesion Types

In each of the 4 other lesion types (hematomas, traumas, tumors, and tubers), negative functional connectivity between lesion locations and voxels in the substantia nigra, globus pallidus internus, and cerebellum was associated with epilepsy (Figure 3A and B; eFigure 12 in Supplement 1). Combining these 4 other lesion types and repeating the lesion network mapping analysis without the ischemic stroke data on a whole-brain level identified almost identical lesion network nodes in the basal ganglia and cerebellum associated with epilepsy (maximum V, 7.3; peak corrected *P* < .001) (eFigure 13A and B in Supplement 1).

### Estimating Risk of Lesion-Related Epilepsy

Functional connectivity with the lesion network nodes in the basal ganglia and cerebellum (Figure 2C) defines a distributed brain network map of areas at increased or decreased risk of epilepsy (Figure 4A). As such, intersection of lesions on this network map provides a convenient tool to visualize epilepsy risk based on lesion location (Figure 4B and C). Functional connectivity between lesion locations from the discovery data set (ischemic stroke data) to the lesion network nodes derived from the validation data sets (other lesion types) was significantly associated with poststroke epilepsy (odds ratio [OR], 2.82; 95% CI, 2.02-4.10; *P* < .001). We repeated this leave-one–data set–out analysis 5 times and found that functional connectivity between lesion locations (from the left-out data set) and the lesion network nodes (derived from the other 4 data sets; eFigure 13C in Supplement 1) was consistently associated with epilepsy across different lesion types (OR, 2.85; 95% CI = 2.23-3.69; *P* < .001). This result remained significant after controlling for lesion volume (adjusted OR [aOR], 2.66; 95% CI, 2.04-3.53; *P* < .001) and damage to the cortex, subcortex, and middle cerebral artery territory (aOR, 2.33; 95% CI, 1.75-3.14; *P* < .001).

Stratifying lesions into categories of high, moderate, and low lesion connectivity to the basal ganglia and cerebellum showed a significant difference in the proportion of epilepsy across categories, both in ischemic stroke (χ^2^, 22.5; *P* < .001) and across all lesion types (χ^2^, 205.3; *P* < .001) (Figure 4D). Results were similar using a receiver operating characteristic analysis that was independent of risk group cutoffs (eFigure 14 Supplement 1) and whether we stratified patients into risk groups within each lesion type or across all lesion types (eFigure 15 in Supplement 1).

### Therapeutic Relevance for DBS

Patients with drug-resistant focal epilepsy had DBS electrodes placed in the anterior thalamus, but the exact placement of the electrode and clinical outcome varied from patient to patient (Figure 5A). Functional connectivity of each patient’s stimulation site (Figure 5B) to the lesion network nodes in the basal ganglia and cerebellum (Figure 2C) was correlated with an improvement in seizure frequency after DBS (*r*, 0.63; *P* < .001) (Figure 5C). Results were similar after controlling for stimulation amplitude (*r*, 0.54; *P* < .001) or volume (*r*, 0.51; *P* = .002). DBS parameters were not significantly correlated with seizure frequency and results were robust to exclusion of an outlier (eFigure 16 in Supplement 1). A voxel-based analysis found that improvement in seizure frequency after DBS was associated with more positive functional connectivity of the patient’s stimulation site to voxels in the substantia nigra, globus pallidus internus, and cerebellum (maximum V, 5.7; peak corrected *P* < .005) (Figure 5D). These same nodes remained significant using a whole-brain analysis (eFigure 17 in Supplement 1).

## Discussion

In this study, lesion locations related to epilepsy mapped to a specific human brain network defined by negative functional connectivity to the basal ganglia and cerebellum. This distributed brain network differentiated lesion locations at increased or decreased risk of epilepsy across different lesion types. Thalamic DBS sites that improve seizure control in drug resistant epilepsy were positively connected to this same network. These findings are potentially relevant for estimating epilepsy risk based on lesion location, understanding lesion-related epilepsy, and improving brain stimulation treatments for epilepsy.

### Relevance for Estimating Epilepsy Risk Based on Lesion Location

The ability to predict which patients with stroke or other lesions are at highest risk of epilepsy could help guide inclusion criteria for antiepileptogenic trials, antiseizure treatment decisions, and patient counseling.^3^ Consistent with previous studies, we found that larger lesions and more damage to the cortex was associated with an increased risk of epilepsy while damage to subcortex was associated with a decreased risk.^3,19,37,38^ However, traditional lesion location mapping was not able to identify an association with damage to any specific brain region or voxel. Rather, lesions associated with epilepsy occurred in multiple heterogenous locations spanning different lobes and vascular territories. In this situation traditional lesion location mapping may require very large cohort sizes to detect neuroanatomical associations.^39^

Despite this heterogeneity, lesion locations associated with epilepsy fell within a specific brain network. Specifically, negative functional connectivity between lesion locations and regions in the basal ganglia and cerebellum was independently associated with epilepsy across 5 different lesion etiologies and data sets. As such, functional connectivity with these subcortical regions defines a distributed brain network map of locations with increased or decreased risk of epilepsy (Figure 4C). This network includes individual brain regions associated with epilepsy in prior lesion mapping studies.^19,30,37,40,41,42^ This network map might be used to better assess epilepsy risk based on lesion location as opposed to individual brain regions, could help inform prognostic models across different lesion etiologies, and may reconcile heterogeneous results across earlier studies. Future prospective work is needed to test whether connectivity can be combined with other variables to improve predictive models.

### Relevance for Understanding Lesion-Related Epilepsy

While epilepsy is often considered a cortical disease and epileptogenesis likely occurs at the lesion location,^2^ our results suggest that connectivity to subcortical nodes may help explain why epilepsy occurs with some lesion locations but not others. Specifically, our results implicate functional connectivity to the basal ganglia and cerebellum, regions which feature prominently in animal model research on seizure modulation.^43,44,45,46,47,48^

Prior work suggests the basal ganglia and cerebellum may act like a “common pathway,”^49,50^ “endogenous control system”^44,45^ or “brake”^51^ of seizures. Lesions, electrical stimulation, and optogenetic modulation of the basal ganglia and cerebellum consistently reduce or terminate seizures in different animal models of epilepsy.^52,53,54,55^ It has been suggested that overt clinical seizures may thus depend on both an epileptogenic focus and a compromised inhibitory control mechanism.^44^ This hypothesis of inhibitory control to suppress or even prevent seizures has seen renewed interest,^56^ as it may help explain why patients with epilepsy do not continuously seize,^57^ why only some seizures generalize,^58,59^ and why seizures stop.^47^ However, where this inhibitory control network might be localized in the human brain has remained a debate and whether it plays a role in epileptogenesis is unknown. Here, we find that lesions causing epilepsy were more negatively connected (ie, *anticorrelated*^60^) to the basal ganglia and cerebellum, which means that when the functional magnetic resonance signal at the lesion location goes up, the functional magnetic resonance signal in the basal ganglia and cerebellum goes down and vice versa.^11,23,60,61^ One potential interpretation is that lesions may have a diaschisislike^62^ effect on these subcortical regions, leading to a general loss of cortical inhibition and seizures. Another potential interpretation is that functional connectivity to the basal ganglia and cerebellum defines the topography of brain regions with more or less intrinsic susceptibility to epilepsy.^63,64^ Although testing mechanistic interpretations of our findings requires future work, our results suggest a link between animal studies on seizure modulation and the location of lesions related to epilepsy in humans.

More broadly, our results support the notion of lesion-related epilepsy as a network disorder.^65,66,67^ Our findings do not preclude an important role for individual epilepsy networks that may differ between patients with focal epilepsy, but rather suggest the coexistence of a common network across different types of focal epilepsy in humans, as previously proposed in animal models of epilepsy.^45,49,50,68^

### Relevance for Brain Stimulation Treatments in Epilepsy

These findings may have therapeutic implications for improving brain stimulation treatments for epilepsy.^9^ Specifically, our results suggest that the antiseizure effects of thalamic DBS may depend on positive connectivity between the stimulation site and a brain network functionally connected to the basal ganglia and cerebellum. This opposing direction of connectivity compared to lesions is consistent with previous findings in depression and the clinical effects (lesions cause seizures while DBS improves seizures).^12^ The finding that brain lesions causing a specific symptom (eg, seizures) are connected to the same network as DBS sites modulating that symptom is in line with previous findings in Parkinson disease, depression, tremor, tics, and addiction.^6,12,13,69^ Convergence across lesions and brain stimulation sites can provide stronger support for network localization than results based on one modality alone, a method termed *convergent causal mapping*.^4,12^

Furthermore, our findings are consistent with the hypothesis that DBS reduces seizures through modulation of brain networks and might help explain why thalamic DBS is effective across different types of focal epilepsy.^70,71,72^ Our results are also in line with evidence from other disorders that clinical effects of DBS depend on connectivity between the stimulation site and remote brain regions.^35^ As such, connectivity to this network might be used to guide DBS (re)programming or even to refine neurosurgical targeting for the treatment of patients with drug-resistant epilepsy. Beyond thalamic DBS, there is some limited evidence that directly targeting the basal ganglia or cerebellum with neuromodulation could have therapeutic value in patients with epilepsy.^73,74,75,76,77,78,79,80^ The specific network topography identified here may help guide future efforts investigating these targets.

### Strengths and Limitations

To our knowledge, this is the first study to examine the association between lesion connectivity and epilepsy across different lesion etiologies. Strengths include validation in 4 independent lesion cohorts with different lesion types, robustness of results to leave-one–data set–out analysis and multiple control analyses, and therapeutic relevance based on convergence with results from an independent DBS data set.

There are several limitations to this study. First, the brain network identified here was derived from focal brain lesions. It remains unknown whether our results are relevant for other etiologies of focal epilepsy, mesial temporal lobe epilepsy, or generalized epilepsy. Second, lesion network mapping uses functional connectivity data from healthy participants to estimate the connectivity of the lesion location in the average human brain.^11^ However, functional connectivity may be altered in individuals with brain lesions or epilepsy, and these alterations can change over time. Prior studies using an age-matched, disease- or patient-specific connectome led to similar lesion- and DBS-network mapping results,^23,35,81,82,83^ but our results in epilepsy remain to be tested in this manner. Third, our stroke control cohorts from the discovery data set were not explicitly tested for epilepsy, which may lead to an underestimate of the effect size in our risk stratification (Figure 4D). Furthermore, this limitation would bias against finding group differences and was not present in the 4 validation data sets, which showed similar connectivity findings. Fourth, due to the retrospective design and data availability, we could not control for variables such as stroke severity or etiology, seizure frequency, subtle structural abnormalities, or predisposing genetic factors. Similarly, small errors or inconsistencies across data sets in lesion tracing and atlas registration are to be expected. However, these limitations should all introduce noise, biasing us against the present converging findings. Fifth, our study highlights common network connections across different lesion types associated with epilepsy, but this does not preclude potentially important differences between lesion etiologies. Sixth, any clinical implications should be interpreted with caution, as our study was based solely on retrospective analyses of existing data sets. Future prospective studies are needed to determine if this network can be used as a clinical tool for prognosis or treatment of epilepsy.

## Conclusions

In this study, lesion-related epilepsy mapped to a human brain network, which could help identify patients at risk for epilepsy after a brain lesion and guide brain stimulation therapies.

## Notes

Supplement 1. eMethods eTable 1. Patient demographics of the discovery dataset eTable 2. Patient demographics of the validation datasets eTable 3. Patient demographics of the DBS dataset eTable 4. Lesion distribution eFigure 1. Lesion network mapping methods flowchart eFigure 2. Lesion overlap eFigure 3. Distribution of lesion damage to the cortex, subcortex, lobes and vascular territories, with and without correcting for lesion volume eFigure 4. Distribution of lesion damage to the mesial temporal lobe with and without controlling for lesion volume eFigure 5. Lesion network mapping control and subgroup analyses eFigure 6. Lesion network mapping results are similar in subgroups of patients with different antiseizure drugs eFigure 7. Lesion network mapping results with subgroups matched for lesion volume and cortical or subcortical damage eFigure 8. Temporal signal to noise ratio of different brain regions in the functional connectome eFigure 9. Statistical mediation analyses eFigure 10. Voxel-based lesion symptom mapping eFigure 11. Lesion network mapping results using a structural connectome eFigure 12. Lesion network mapping results of each individual dataset and lesion etiology eFigure 13. Whole-brain lesion network mapping results in discovery and validation datasets eFigure 14. Receiver operating characteristics (ROC) curves eFigure 15. Proportion of epilepsy in categories based on lesion connectivity eFigure 16. Correlation between DBS site connectivity and clinical outcome eFigure 17. Whole-brain DBS network mapping results

Supplement 2. Data sharing statement
