# Supplementary material for: Mapping Lesion-Related Epilepsy to a Human Brain Network
Source: JAMA Neurol. 2023 Jul 3;80(9):891–902. doi: 10.1001/jamaneurol.2023.1988 (PMC10318550; doi:10.1001/jamaneurol.2023.1988)
Supplement: Supplement 1. — eMethods eTable 1. Patient demographics of the discovery dataset eTable 2. Patient demographics of the validation datasets eTable 3. Patient demographics of the DBS dataset eTable 4. Lesion distribution eFigure 1. Lesion network mapping methods flowchart eFigure 2. Lesion overlap eFigure 3. Distribution of lesion damage to the cortex, subcortex, lobes and vascular territories, with and without correcting for lesion volume eFigure 4. Distribution of lesion damage to the mesial temporal lobe with and without controlling for lesion volume eFigure 5. Lesion network mapping control and subgroup analyses eFigure 6. Lesion network mapping results are similar in subgroups of patients with different antiseizure drugs eFigure 7. Lesion network mapping results with subgroups matched for lesion volume and cortical or subcortical damage eFigure 8. Temporal signal to noise ratio of different brain regions in the functional connectome eFigure 9. Statistical mediation analyses eFigure 10. Voxel-based lesion symptom mapping eFigure 11. Lesion network mapping results using a structural connectome eFigure 12. Lesion network mapping results of each individual dataset and lesion etiology eFigure 13. Whole-brain lesion network mapping results in discovery and validation datasets eFigure 14. Receiver operating characteristics (ROC) curves eFigure 15. Proportion of epilepsy in categories based on lesion connectivity eFigure 16. Correlation between DBS site connectivity and clinical outcome eFigure 17. Whole-brain DBS network mapping results [file jamaneurol-e231988-s001.pdf]

Supplemental Online Content

Schaper FLWVJ, Nordberg J, Cohen AL, et al. Mapping lesion-related epilepsy to a human brain network. *JAMA Neurol*. Published online July 3, 2023. doi:10.1001/jamaneurol.2023.1988

eMethods

eTable 1. Patient demographics of the discovery dataset

eTable 2. Patient demographics of the validation datasets

eTable 3. Patient demographics of the DBS dataset

eTable 4. Lesion distribution

eFigure 1. Lesion network mapping methods flowchart

eFigure 2. Lesion overlap

eFigure 3. Distribution of lesion damage to the cortex, subcortex, lobes and vascular territories, with and without correcting for lesion volume

eFigure 4. Distribution of lesion damage to the mesial temporal lobe with and without controlling for lesion volume

eFigure 5. Lesion network mapping control and subgroup analyses

eFigure 6. Lesion network mapping results are similar in subgroups of patients with different antiseizure drugs

eFigure 7. Lesion network mapping results with subgroups matched for lesion volume and cortical or subcortical damage

eFigure 8. Temporal signal to noise ratio of different brain regions in the functional connectome

eFigure 9. Statistical mediation analyses

eFigure 10. Voxel-based lesion symptom mapping

eFigure 11. Lesion network mapping results using a structural connectome

eFigure 12. Lesion network mapping results of each individual dataset and lesion etiology.

© 2023 Schaper FLWVJ et al. *JAMA Neurol.*

eFigure 13. Whole-brain lesion network mapping results in discovery and validation datasets
eFigure 14. Receiver operating characteristics (ROC) curves
eFigure 15. Proportion of epilepsy in categories based on lesion connectivity
eFigure 16. Correlation between DBS site connectivity and clinical outcome
eFigure 17. Whole-brain DBS network mapping results.

This supplemental material has been provided by the authors to give readers additional information about their work.

© 2023 Schaper FLWVJ et al. *JAMA Neurol.*

eMethods

Stroke patients and brain lesions

Stroke patients

We retrospectively studied lesion locations from 76 patients with new onset post-stroke epilepsy (ischemia) that were part of a previous study. A description of this dataset has been posted on medRxiv[1], but this paper does not include any of the analyses or results presented here. In this previous study, a systematic hospital wide medical record search was performed to select patients with an age ≥ 18 years old, a brain MRI with a visible ischemic stroke, and a diagnosis of symptomatic epilepsy made between 2004 and 2017 at the Turku University Hospital, Turku, Finland. A diagnosis of post-stroke epilepsy was made by retrospective review of diagnosis codes, clinical charts, semiology, EEG and neuroimaging. All included patients had: (i) a diagnosis of new onset epilepsy associated with ischemic stroke according to current ILAE criteria, including at least two unprovoked seizures occurring more than 24 hours apart, more than seven days after stroke onset (i.e. late seizures), (ii) brain MRI obtained three months prior or after epilepsy diagnosis (iii) one or more focal ischemic stroke lesions visible on MRI, (iv) no other brain lesions or structural abnormalities, and (v) no history of seizures prior to their stroke. Patients with a single seizure, evoked seizures, or only seizures within seven days after stroke onset (i.e., early seizures) were excluded. Patients with unclear etiology, secondary brain lesions after their first stroke or other likely causes of epilepsy such as intracranial surgery or electrolyte disturbances were excluded. In total, 76 patients fulfilled all criteria and were included in the study.

Brain lesions

Lesion locations were previously manually segmented on high-resolution patient-specific MRI scans using FSLeyes[2] software (https://fsl.fmrib.ox.ac.uk/fsl/fslwiki/FSLeyes). All slices in the coronal, sagittal and horizontal planes were examined and the lesion location was segmented in all three planes on T1 or T2 weighted sequences. Lesioned voxels were assigned a 1 and non-lesioned voxels were assigned a 0, resulting in a three-dimensional binary lesion mask. Each patient's lesion mask was subsequently spatially normalized to a common atlas (Montreal Neurological Institute (MNI) space, Figure 1A) using FMRIB's linear image registration tool (FLIRT) implemented in FSL. Linear registration was used as opposed to non-linear registration because structural brain lesions such as stroke may affect the gross brain anatomy leading to bias in lesion location.[3,4]

To control for the normal distribution of stroke lesions, two independent and previously published datasets of consecutive stroke patients with lesion locations not associated with epilepsy were used as controls (n = 135[5], n = 490[6]) as in our prior work.[16,20] The first control dataset included 135 lesion masks, part of the Washington University Stroke Project[5]. The second control dataset included 490 lesion masks, part of the Genes Associated with Stroke Risk and Outcomes Study (GASROS) collected at Massachusetts General Hospital[6]. Investigating the relationship between lesion location and epilepsy was not a goal of these studies and therefore information on whether patients developed post-stroke epilepsy was not collected. The prevalence of epilepsy in these control cohorts is expected to be 5-10%,[7] much less than the 100% in our post-stroke epilepsy cohort. However, the presence of some patients with epilepsy in these control cohorts may bias us against finding significant differences. See eTable 1 for patient demographics (discovery dataset).

Lesion location mapping

Traditional lesion location mapping methods were used to test whether lesions associated with post-stroke epilepsy map to a common brain region.

A priori region of interest analysis

First, we assessed the maximum lesion overlap across any voxel in the whole brain. Second, we calculated the lesion volume and assessed the overlap (or damage) of each lesion to masks of a priori regions of interest (ROIs): the cerebral cortex, subcortex, cortical lobes (including mesial temporal lobe), and vascular territories. The cerebral cortex mask was defined by combining all cortical lobe masks from the Harvard-Oxford Cortical Atlas[8] (masks were thresholded and binarized at 25% probability). The subcortex mask was defined by subtracting the cerebral cortex mask from the MNI brain mask. The lobar masks of the frontal, parietal, occipital, and temporal lobes were defined by the MNI Structural Atlas[9], as this atlas is more liberal and includes the adjacent white matter in contrast to only the gray matter included in the Harvard Oxford Cortical Atlas[8]. The mesial temporal lobe mask was defined by adding and binarizing

© 2023 Schaper FLWVJ et al. *JAMA Neurol.*

the hippocampus and amygdala masks from the Harvard-Oxford SubCortical Atlas[8] (masks were thresholded and binarized at 25% probability). The vascular territories masks were defined by the 'Vascular Territory template and atlas in MNI space'.[10] Damage to these a priori ROIs was quantified by calculating the number of lesioned voxels intersecting with each mask and dividing by the number of voxels in each brain region mask, resulting in the percentage of the brain region damaged by the lesion. Association between percentage damage to these regions and epilepsy diagnosis was analyzed with an Aspin-Welch test, assessed using permutations, while controlling for lesion volume as a covariate (i.e. nuisance variable) and correcting for multiple testing. A two-tailed family wise error corrected p-value < 0.05 was considered significant. The Aspin-Welch test, implemented in the tool PALM (Permutation Analysis of Linear Models, https://fsl.fmrib.ox.ac.uk/fsl/fslwiki/PALM)[11], does not assume identical variances, thus accommodating different distributions between the groups.

Voxel-based lesion symptom mapping analysis
To identify any lesioned brain voxels associated with epilepsy, we used univariate voxel-based lesion-symptom mapping (VLSM) in NiiStat (https://github.com/neurolabusc/NiiStat)[12,13] and multivariate VLSM[14,15] in the SVR-LSM toolbox (https://github.com/atdemarco/svrlsmgui)[16].

NiiStat is a Matlab software package that performs univariate VLSM, with a general linear regression model and permutation test, controlling for covariates. We limited our analysis to voxels occurring in at least 5% of lesions, assessed with Freedman-Lane permutations (the default setting of 2000 permutations was used), while controlling for lesion volume as a covariate and correcting for multiple testing. These parameters were chosen based on published best-practice recommendations[12,13,17]. A two-tailed family wise error corrected p-value < 0.05 was considered significant. SVR-LSM is a Matlab software package that performs multivariate voxel- and cluster-based LSM with a machine learning regression, termed the support vector regression (SVR). In contrast to univariate VLSM, which considers neighboring voxels as independent. Multivariate SVR-VLSM simultaneously considers many voxels at once when determining whether damaged brain regions contribute to behavioral deficits[15,16]. These multivariate LSM approaches can identify complex dependences that traditional univariate VLSM approaches cannot. In line with the univariate VLSM analysis in NiiStat, we limited our analysis for multivariate VLSM to voxels occurring in at least 5% of lesions, assessed with permutations (default setting of 10,000 permutations was used), while controlling for lesion volume using the standard "direct total lesion volume control" (dTLVC) approach and correcting for multiple testing. A two-tailed family wise error corrected p-value < 0.005 was considered significant. These parameters were chosen based on published best-practice recommendations[14–16].

Additionally, we explored univariate and multivariate VLSM results using liberal statistical cutoffs and assessed their alignment with our findings from lesion-network mapping by overlapping statistically significant VLSM voxels with lesion network mapping results.

Lesion network mapping

Lesion network mapping combines the lesion location associated with a specific symptom or neuropsychiatric disease with the human brain connectome to estimate the brain network connected to each lesion location. In this method, the lesion location is commonly derived from the structural brain images of a patient, while the human brain connectome is derived from functional MRI (fMRI) data of healthy participants. The term "network" in lesion network mapping refers to "voxels that show a temporal correlation in spontaneous fMRI fluctuations with the lesion location".[18]

Lesion network mapping was performed to test whether lesions associated with post-stroke epilepsy map to a specific brain network. As described previously,[19,20] we performed seed-based functional connectivity analyses between each lesion location and all other brain voxels using the resting state functional connectivity data (2 × 2 × 2 mm resolution) from 1000 healthy participants (human brain connectome: https://dataverse.harvard.edu/dataverse/GSP).[21,22] This process results in a lesion network for each lesion location (Figure 2A-B, eFigure 1). More details are described below.

The human brain connectome
Resting state functional connectivity was obtained from 1000 healthy participants, using a high-resolution 3T MRI scanner in the 'Open Access' Brain Genomics Superstruct Project (GSP) (https://dataverse.harvard.edu/dataverse/GSP). MRI data acquisition was performed at Harvard University and Massachusetts General Hospital using a 12-channel phased-array head coil. Structural data included a high-resolution (1.2mm isotropic) multi-echo T1-weighted magnetization-prepared gradient-echo image. Functional imaging data with whole-brain coverage in the resting state (still, stay awake, and keep their eyes open while blinking normally)

© 2023 Schaper FLWVJ et al. *JAMA Neurol.*

were acquired on a gradient-echo echo-planar imaging (EPI) sequence sensitive to blood oxygenation level-dependent (BOLD) contrast. BOLD runs consisted of 47 interleaved slices, with 124 measurements collected for each BOLD run (TR = 3000 msec; 4 initial TRs collected to allow for T1-stablization and 120 valid measurements). One or two BOLD runs were acquired per subject (72.6% of sessions included two runs). Preprocessing of these scans has been fully described elsewhere,[21,22] and included regression of noise variables derived from motion, CSF, white matter, and the global signal.

Computing lesion networks from lesion locations using the human brain connectome

To compute a lesion-network map, each lesion location was used as a seed in resting state functional connectivity analysis of the data collected from each of the 1000 participants included in the human connectome (see methods flowchart in eFigure 1). The time series for voxels within the lesion location were correlated with the time series from all other brain voxels and results were statistically combined across the 1000 participants to create a voxel-based T-map, as described before.[19,20] The resulting T-map, also termed a lesion-network map, represents the strength and consistency of functional connectivity for each lesion location between all other brain voxels (Figure 2A-B). Positive functional connectivity (warm colors) refers to a positive correlation of blood-oxygen-level-dependent (BOLD) timeseries between the lesion location and all other brain voxels, while negative functional connectivity (cool colors) refers to a negative correlation (i.e. anticorrelation) of BOLD timeseries between lesion location and all other brain voxels. In other words, when the BOLD activity in the lesion location goes up, BOLD activity will also go up in the brain regions positively correlated to the lesion location but will go down in the regions negatively correlated and vice versa.

Identifying the functional connections associated with epilepsy

To identify functional connections associated with epilepsy, we performed a whole-brain voxel-based permutation test using the software Permutation Analysis of Linear Models (PALM) (https://fsl.fmrib.ox.ac.uk/fsl/fslwiki/PALM), while controlling for lesion volume as a covariate and correcting for multiple testing, in line with previous lesion network mapping studies.[23,24] We used an Aspin-Welch test at each voxel, allowing for unequal variance between groups, 2000 permutation and the p-values were computed using a generalized Pareto distribution fitted to the tail of the permutation distribution.[11] The resulting output is a spatial map of voxels more positively or negatively connected ("anticorrelated)[25] to lesion locations associated with epilepsy (Figure 2C). A two-tailed family wise error corrected p-value < 0.05 was considered significant, however higher statistical thresholds were often used to highlight the most significant findings (see Figure legends).

Consistency of results with and without global signal regression

It is worth highlighting that our normative connectome (GSP) was preprocessed using global signal regression, which greatly reduces the influence of non-specific variance, but may complicate interpretation of negative correlations.[26,27] To ensure our results were similar between connectome preprocessing methods, we repeated our lesion network mapping analysis using a different 100-subject functional connectivity dataset generated without using global signal regression, similar to prior studies.[19,28] Resting state functional MRI data were processed using the aCompCor strategy as implemented in the Conn Toolbox (www.nitrc.org/projects/conn),[29,30] which includes regression of noise variables derived from motion, CSF, and white matter, but not the global signal. All settings for preprocessing and regression were kept as default/recommended.

Consistency of results after controlling for covariates

To test whether lesion network mapping results were similar after controlling for variables such as age, sex, acute seizyre and lesion volume or known epilepsy risk factors (damage to the cortex, subcortex and MCA territory[7]), we repeated our lesion network mapping analyses including these variables as a covariate (nuisance variables) in the PALM design matrix. To ensure our results were similar across multiple stroke lesion datasets, seizure type (focal only or focal to bilateral tonic clonic), delay to first seizure after stroke (within or after 6 months), EEG abnormalities (epileptiform / slowing or only clinical evidence of seizures), and patients using different antiseizure drugs, we repeated our lesion network mapping analysis in PALM for these subgroups of patients.

Consistency of results in a matched subgroup analysis

To test whether lesion network mapping results were similar comparing epilepsy lesions to a subgroup of controls that were matched for lesion volume and damage to the cortex and subcortex, we used propensity score matching (https://github.com/kosukeimai/MatchIt)[31–34]. Propensity score matching is a validated method used to account for confounds in observational studies which allows one to precisely generate two matched groups that are equivalent

across multiple confounds/covariates except for the independent variable of interest. Specifically, a propensity score was calculated for each subject by fitting a logistic regression model where the response variable is group membership (epilepsy vs. control) and the explanatory variables are the confounds (damage to the cortex and subcortex). A matched control subject (n = 76 of 625 original controls) for each epilepsy subject (n = 76) was selected, based on the closest propensity score. We performed this matched group analyses twice, first matching groups based on lesion volume (model: epilepsy ~ lesion volume), and second, matching groups on damage to the cortex and subcortex (model: epilepsy ~ percentage damage to the cortex + percentage damage to the subcortex). After matching groups, we performed a lesion-network mapping analysis in PALM on this subset. We tested whether the primary findings from our full ischemic stroke cohort persisted in this smaller matched subset.

Mediation analysis to assess the relationship between independent and dependent variables
To assess the relationship between lesion connectivity, lesion volume, damage to the cortex and subcortex, and epilepsy diagnosis, we performed statistical mediation analyses using the *lavaan* R package (https://github.com/yrosseel/lavaan.git),[35] with the recommended 5000 bootstrap samples to calculate significance of the indirect pathway via confidence intervals.

Lesion network mapping using a structural connectome
We repeated our lesion network mapping analysis in PALM using structural connectivity derived from BCBtoolkit, a widely implemented software package to assess white matter tract disconnection of brain lesions. We used a similar method as described in one of our previous papers.[36]

First, we performed lesion network mapping analysis using structural connectivity instead of functional connectivity. We calculated the probability of a given voxel to be disconnected on a whole-brain level, resulting in a structural lesion network for each lesion location (n=701 of which 76 with epilepsy). We identified the structural connections associated with epilepsy with PALM, using an identical method as used in our functional connectivity analyses. Second, we explored convergence / divergence between structural and functional lesion network mapping by testing whether disconnection of structural connections to our functional lesion network nodes differed between epilepsy and control lesions. Specifically, we computed structural connectivity with each lesion location, then computed the overlap of this structural connectivity map with our functional lesion network nodes ("disconnectome score"). Finally, disconnectome scores of lesions associated with epilepsy or control were compared using an Aspin-Welch test.

Generalizability across different lesion types

Other lesion type datasets
To test for generalizability, we studied four validation datasets of other lesion etiologies: brain hematoma locations in patients with hemorrhagic stroke (n = 320, 7% with epilepsy),[37] brain injury locations in Vietnam war veterans with penetrating head trauma in (n = 197, 44% with epilepsy),[38] brain tumor locations in patients with glioblastoma multiforme (n = 132, 46% with epilepsy),[39] and cortical tuber locations in children with Tuberous Sclerosis Complex (n = 123, 81% with epilepsy).[40] These lesion datasets were selected for inclusion in the current study because 1) they were used in prior publications relating lesion locations to epilepsy, 2) lesion locations were made available to us for analyses, and 3) a control dataset of similar lesions not associated with epilepsy was also available. All datasets meeting these criteria were included in the current study. No datasets were included or excluded after analysis. For each dataset, we used either a validated segmentation algorithm (https://github.com/msharrock/deepbleed)[41] to outline the lesion locations (hemorrhagic stroke) or used the previously published lesion outlines (penetrating heat trauma, glioblastoma multiforme, tuberous sclerosis complex) to avoid any potential risk of bias. See eTable 2 for patient demographics and details on each dataset (validation datasets).

Generalizability of post-stroke epilepsy network findings to other lesion types
Using the connections derived from ischemic stroke lesions as an a priori region of interest (ROI, Figure 2A-B), we tested the hypothesis that each of the other lesion types would show similar connectivity differences between epilepsy and control lesions. We repeated the same voxel-based permutation test in PALM controlling for lesion volume that we used in our primary analysis but limited our search space to our a priori ROI derived from the ischemic stroke data and used a more liberal statistical cutoff given the reduced sample size (one-tailed false discovery rate corrected p-value < 0.05). Note that we have previously reported on a subset of these connections in tubers associated with infantile

spasms[54] (a specific infantile epilepsy syndrome) but are extending these results here to epilepsy diagnosis and other lesion types.

Estimating risk of lesion-related epilepsy

A brain network for lesion-related epilepsy

Using the lesion network nodes derived from post-stroke epilepsy (Figure 2C), we computed a distributed brain network map with regions of increased and decreased risk of epilepsy (Figure 4A). The whole-brain functional connectivity of each lesion network node (GPi, SN, or cerebellum) was computed separately by running these nodes as a weighted seed in the same human connectome used for the lesion network mapping analysis (GSP). The whole-brain functional connectivity maps of these three separate nodes were then averaged into one whole-brain map and inverted so that lesion locations at increased risk of epilepsy are represented in warm colors and lesion locations at decreased risk of epilepsy are represented in cool colors (Figure 4A). Intersection of lesions on this distributed brain network map thus provides a convenient tool to visualize epilepsy risk based on lesion location (Figure 4B and C)

Leave-one-dataset-out analysis

For the leave-one-dataset-out-analysis, the five lesion types were defined as five datasets and we iteratively used four datasets to test the left-out dataset. We combined these four datasets, and identified the lesion network nodes significantly associated with epilepsy across different lesion types (leaving out ischemic stroke lesions). This whole-brain PALM analysis was identical to our primary lesion network mapping analysis in ischemic stroke but controlled for lesion type to identify the connections specific to epilepsy across different lesion types, while correcting for lesion volume as a covariate and correcting for multiple testing. Significance was assessed with permutations, and controlled for batch effects using exchangeability blocks to permute within each lesion dataset / type.[42] As in our primary analysis in ischemic stroke, a two-tailed family wise error corrected p-value < 0.05 was considered significant. Next, we computed the functional connectivity between each lesion from the left-out dataset to the lesion network nodes generated from the other four datasets, by correlating the average functional MRI (fMRI) signal between these two regions (ROI-to-ROI connectivity), using the same normative connectome (n=1000) used for the lesion network mapping analysis. This leave-one-dataset-out process was repeated five times, each time leaving out a different lesion type. To evaluate the potential prognostic relevance of this network, association between post-stroke epilepsy and this out-of-sample lesion connectivity value was tested using logistic regression, controlling for lesion volume and known epilepsy risk factors (damage to the cortex, subcortex, and MCA territory).

Estimating risk of lesion-related epilepsy

These lesion connectivity values were then used to stratify patients into three risk categories similar to previous work[24]: high-fc (functional connectivity one SD above the mean), low-fc (functional connectivity one SD below the mean) and moderate-fc (patients in between the high and low risk groups). A Chi-squared test was performed to compare the proportion of epilepsy across the different risk groups. To ensure our results were not biased by lesion type, we repeated this risk stratification by categorizing subjects within each lesion type instead of across lesion types. To ensure results were independent of our risk group cutoffs, we repeated this analysis using receiver operating characteristics (ROC) and computed the area under the curve (AUC).

Power analysis for risk stratification

The achieved power was calculated using the software G*Power[43]. Power was 1 for risk stratification of all datasets (Figure 4D left panel) assuming a Chi2= 205.3, n=1473, df = 2, $\alpha = 0.05$, and Cramer's V effect size = 0.264. Power was 0.86 for risk stratification of post-stroke epilepsy (Figure 4D right panel), assuming a Chi2= 22.5, n=701, df = 2, $\alpha = 0.05$, and Cramer's V effect size = 0.127.

Therapeutic relevance for deep brain stimulation

DBS patients

To evaluate the potential therapeutic relevance of this network, we analyzed the DBS electrode locations of 30 patients with drug resistant epilepsy who received DBS of the anterior nucleus of the thalamus (ANT), the FDA approved DBS target for focal epilepsy.[44] Patient demographics are presented in eTable 3. We tested whether improved clinical outcome was associated with DBS sites that are more connected to our lesion network nodes. Clinical outcome was measured by the percentage of change in seizure frequency, obtained from standard seizure diaries.

© 2023 Schaper FLWVJ et al. *JAMA Neurol.*

DBS electrode localization and computing stimulation sites

Each patient's DBS electrode locations and stimulation sites were localized in MNI space using Lead-DBS (https://www.lead-dbs.org)[45] and patient specific stimulation parameters, similar to previous studies.[46] Briefly, pre-operative T1 / T2 MRI sequences and post-operative MRI / CT images were linearly co-registered using SPM (https://www.fil.ion.ucl.ac.uk/spm/software/spm12/[47]). Co-registration was further refined using the 'brainshift correction[48]' option and images were normalized to MNI space using the Advanced Normalization Tool (http://stnava.github.io/ANTs/[49]). DBS electrode trajectories and contacts were automatically pre-localized and manually refined using Lead-DBS. Each patient's stimulation site (also termed volume of activated tissue or VAT) was modeled in MNI space using patient specific stimulation settings and a finite element approach in an adapted version of the Fieldtrip/Simbio pipeline included in Lead-DBS (http://www.fieldtriptoolbox.org/; https://www.mrt.unijena.de/simbio/[50]).

Calculating DBS site connectivity to lesion network nodes

We then calculated the functional connectivity of each patient's stimulation site to the lesion network nodes (Figure 2C) using ROI-to-ROI connectivity. Functional connectivity between these regions was calculated using the same normative connectome dataset (n=1000) used in the lesion network mapping analysis described above. We tested for correlation between this connectivity value and seizure frequency with a Pearson correlation (r) and permutation testing. To control for the effect of stimulation amplitude, we repeated this correlation using DBS amplitude (V) and VAT volume (voxels) as a covariate. To test whether these results were robust to outliers, we repeated this correlation excluding an outlier with worsened seizure control after DBS. DBS parameters such as frequency (Hz) and pulse width (μs) were also correlated with clinical outcome. The achieved power for DBS site connectivity correlation with clinical outcome was 0.97, assuming a two-tailed $r(H0) = 0$, $r(H1) = -0.63$, $n = 30$, $\alpha = 0.05$.

DBS network mapping analysis

We performed a voxel-based DBS network mapping analysis using PALM to identify connections significantly associated with DBS response within the a priori ROI of the lesion network nodes derived from the ischemic stroke data (Figure 2A-B). A two-tailed false discovery rate corrected p-value < 0.05 was considered significant, similar to the analyses for voxel-based testing of generalizability to other lesion types (Figure 3). Finally, clusters of connections significantly associated with DBS response outside this a priori ROI were defined using a whole-brain PALM analyses with threshold-free cluster enhancement. A two-tailed family wise error corrected p-value < 0.05 was considered significant.

© 2023 Schaper FLWVJ et al. *JAMA Neurol.*

eTable 1

Discovery data	Post-stroke epilepsy	Control dataset 1	Control dataset 2
**Reference**	Nordberg *et al.* 2021[1]	Corbetta *et al.* 2015[5]	Wu *et al.* 2015[6]
**N**	76	135	490
**Sex (male/female)**	39 / 37	63 / 72	303 / 187
**Age at scan (years)**	61 [14.6]	53.6 [10.8]	65 [14.9]
**Time between stroke and first seizure (days)**	978 [2162]	NA	NA
**Seizure type, n (%)**		NA	NA
Focal seizures only	31 (40.8%)		
Focal to bilateral tonic clonic seizures	45 (59.2%)		
**EEG abnormalities, n (%)**		NA	NA
Normal	23 (30.3%)		
Epileptiform	25 (32.9%)		
Focal slowing	20 (26.3%)		
Unknown	8 (10.5%)		
**Antiepileptic drugs (AEDs), n (%)**		NA	NA
0	2 (2.6%)		
1	66 (86.8%)		
2	7 (9.2%)		
3	1 (1.3%)		
**Brain scan**	MRI	MRI	MRI
**MRI field strength**	1.5 or 3T	3T	1.5 or 3T
**MRI sequence**	T1 and T2	T1, T2 and Flair	DWI
**Lesion locations were available in MNI space**	Yes	Yes	Yes
**Lesion segmentation**	Manual	Manual	Manual
**Follow-up**	6.7 [2.0] years	3 – 12 months	3 – 6 months

eTable 1. Patient demographics of the discovery dataset. Patients with post-stroke epilepsy (ischemia) and two independent consecutive stroke cohorts (control datasets 1 and 2). Values are presented as means and standard deviations [SD], intervals or percentages as appropriate. Note that while descriptions of these lesion datasets have been previously published, all analyses and results are unique to the present paper.

© 2023 Schaper FLWVJ et al. *JAMA Neurol.*

eTable 2

Validation data	Hemorrhagic stroke	Penetrating head trauma	Glioblastoma multiforme	Tuberous Sclerosis Complex
**Reference**	Greef *et al* 2014[37]	Raymont *et al.* 2010[38]	Cayuela *et al.* 2018[39]	Cohen *et al.* 2021[40]
**N total**	320	197	132	123
**N with epilepsy**	23 (7%)	87 (44%)	61 (46%)	100 (81%)
**N without epilepsy**	297 (93%)	110 (56%)	71 (54%)	23 (19%)
**Sex (male/female)**	172 / 148	197 / 0	83 / 49	63 / 51
**Age at scan (years)**	71.1 [13.6]	58.3 [3.1]	60.7 [11.6]	2.66 [0.947]
**Brain scan**	CT	CT	MRI	MRI
**MRI field strength**	NA	NA	1.5T	3T
**MRI sequence**	NA	NA	T1 with contrast	T1. T2 and Flair
**Lesion locations were available in MNI space**	No*	Yes	Yes	Yes
**Lesion segmentation**	Automatic	Manual	Manual	Automatic with manual correction
**Follow-up**	up to 8 years	30 – 35 years	up to 2 years	3 years

eTable 2. Patient demographics of the validation datasets. Values are presented as means and standard deviations [SD] or percentages, as appropriate. Note that while descriptions of these lesion datasets have been previously published, all analyses and results are unique to the present paper. *This previously published study on hemorrhagic stroke locations associated with epilepsy did not outline the precise lesion location, but identified the gross neuroanatomical involvement of each lesion by clinical judgement (cortical or subcortical, lobe, vascular territory). We therefore requested the data of this study and used a validated deep-learning segmentation algorithm (DeepBleed: https://github.com/msharrock/deepbleed) to segment the lesion location and transform the lesions to MNI space.

© 2023 Schaper FLWVJ et al. *JAMA Neurol.*

eTable 3

Subject	Sex	Age at surgery (years)	Epilepsy duration (years)	Seizure-onset zone	Previous resection or VNS	MRI findings	DBS Active contacts		DBS amplitude (V)		DBS frequency (Hz)	DBS pulse width (µs)	Δ Seizure frequency after DBS (%)
							Left	*Right*	*Left*	*Right*	Bilateral	Bilateral	
1	Male	Early 40s	20	Temporal	Left temporal resection	Post-surgery	1-C+	1-C+	6	6	140	90	-100
2	Female	Mid 30s	33	Bilateral temporal	VNS	Mesial temporal sclerosis	1-C+	1-C+	6	6	145	60	-48
3	Male	Mid 60s	51	Left frontal	Left temporal resection and VNS	Post-surgery	0-1+	0-1+	5	5	125	90	-42
4	Male	Mid 40s	23	Right Parietal	VNS	Occipito-temporal cortical dysplasia	2-C+	1-C+	6	6	145	90	-19
5	Male	Late 40s	39	Left temporal	None	Mesial temporal tumor	1-C+	0-C+	5.5	5.5	145	90	-100
6	Male	Mid 30s	13	Bilateral temporal	VNS	Mesial temporal sclerosis	1-C+	1-C+	7	7	145	90	+82
7	Female	Early 30s	12	Bilateral temporal	Left temporal resection and VNS	Post-surgery	1-C+	1-C+	6.2	6.2	90	90	-66
8	Male	Early 40s	30	Frontal	None	Mesial temporal sclerosis	1-C+	1-C+	6	6	145	90	-47
9	Male	Late 30s	29	Multifocal	VNS	MRI-negative	2-C+	1-C+	5	5	145	90	-49
10	Male	Early 40s	29	Bilateral parietal	VNS	Parietal cortical dysplasia	1-C+	1-C+	5.4	5.4	160	60	-70

© 2023 Schaper FLWVJ et al. *JAMA Neurol.*

11	Female	Early 20s	19	Left frontal	VNS	MRI-negative	1-C+	1-C+	2	2	145	90	-100
12	Male	Mid 30s	32	Multifocal	VNS	MRI-negative	1-C+	1-C+	5.5	5.5	140	90	-100
13	Male	Early 20s	14	Multifocal	VNS	Parietal lesion of unknown origin	1-2+	1-2+	3.5	3.5	145	90	+7
14	Male	Early 50s	48	Left frontal	VNS	Frontal cortical dysplasia	2-C+	2-C+	6.15	6.15	145	90	-41
15	Male	Mid 40s	18	Left frontal	Left frontal resection and VNS	Post-surgery	2-C+	2-C+	3	3	145	90	-77
16	Male	Late 20s	20	Parietal	Left parietal resection	Post-surgery	1-C+	1-C+	5.7	5.7	160	90	-83
17	Male	Mid 50s	37	Left temporal	VNS	MRI-negative	0-C+	1-C+	4	4	145	90	-63
18	Male	Early 60s	46	Right frontal	VNS	MRI-negative	2-C+	1-C+	4.5	4.5	145	90	-44
19	Female	Mid 30s	26	Parietal	VNS	MRI-negative	1-C+	0-C+	5.8	5.8	160	90	-89
20	Male	Mid 30s	18	Multifocal	VNS	MRI-negative	0-1+	1-C+	3	5.5	120	90	+7
21	Male	Mid 20s	9	Temporal	VNS	MRI-negative	1-2+	1-2+	3.5	3.5	145	90	-3
22	Male	Early 20s	18	Bilateral temporal	None	MRI-negative	2-C+	1-C+	5	5	145	90	-50
23	Female	Early 50s	31	Bilateral temporal	VNS	MRI-negative	1-C+	1-C+	5.7	5.7	145	90	-58
24	Male	Late 30s	31	Bilateral parieto-occipital	None	Perinatal ischemic injury	0-1+	0-1+	3.5	3.5	160	90	+33
25	Female	Mid 30s	18	Multifocal	VNS	MRI-negative	1-C+	1-C+	3	3	145	90	-67
26	Female	Early 20s	11	Multifocal	None	MRI-negative	1-C+	1-C+	2.5	2.5	145	90	+100

© 2023 Schaper FLWVJ et al. *JAMA Neurol.*

27	Male	Late 50s	40	Bilateral temporal	None	MRI-negative	1-C+	2-C+	5.5	5.5	145	90	-50
28	Male	Early 50s	50	Multifocal	None	MRI-negative	1-C+	1-C+	2.5	2.5	145	90	0
29	Female	Mid 40s	39	Multifocal	None	MRI-negative	1-C+	1-C+	5.9	5.9	145	90	-75
30	Female	Late 40s	22	Bilateral frontal	VNS	MRI-negative	1-C+	1-C+	5	5	145	90	-25

eTable 3. Patient demographics of the DBS dataset. Note that while a description of this DBS dataset (including 20 of these 30 patients) has been previously published[44], all analyses and results are unique to the present paper. *Abbreviations: VNS, vagal nerve stimulation.*

© 2023 Schaper FLWVJ et al. *JAMA Neurol.*

eTable 4

	Epilepsy	Control	Total
**Reference**	Nordberg *et al.* 2021[1]	Corbetta *et al.* 2015[6] and Wu *et al.* 2015[7]	
**Patients, n**	76	625	701
**% Damage to brain region**			
Brain (i.e. lesion volume)	3.4% [4.3]	2.0% [3.5]	2.1% [3.6]
Cortex / Subcortex			
Cortex	3.8% [4.8]	1.7% [3.7]	2.0% [3.9]
Subcortex	3.5% [4.7]	2.6% [4.0]	2.7% [4.1]
Lobes			
Frontal lobe	3.0% [5.3]	1.5% [4.7]	1.6% [4.8]
Occipital lobe	3.1% [6.2]	1.4% [4.4]	1.6% [4.7]
Parietal lobe	4.7% [6.9]	2.1% [4.7]	2.4% [5.0]
Temporal lobe	4.6% [8.5]	1.9% [5.4]	2.2% [5.8]
Mesial temporal lobe	1.7% [4.7]	1.6% [5.2]	1.7% [5.1]
Vascular territories			
Anterior cerebral artery	1.8% [3.4]	1.0% [3.4]	1.1% [3.4]
Middle cerebral artery	5.6% [7.8]	3.1% [6.0]	3.4% [6.3]
Posterior cerebral artery	2.0% [3.7]	1.2% [2.9]	1.3% [3.0]
**Frequency of involvement of brain region, n (%)**			
Cortex / Subcortex			
Cortex	68 (89%)	447 (72%)	515 (74%)
Subcortex	76 (100%)	622 (99%)	698 (99%)
Lobes			
Frontal lobe	45 (59%)	305 (49%)	350 (50%)
Occipital lobe	35 (46%)	187 (30%)	222 (32%)
Parietal lobe	54 (71%)	349 (56%)	403 (57%)
Temporal lobe	46 (61%)	290 (46%)	336 (48%)
Mesial temporal lobe	17 (22%)	166 (27%)	183 (26%)
Vascular territories			
Anterior cerebral artery	54 (71%)	395 (63%)	449 (64%)
Middle cerebral artery	72 (95%)	522 (84%)	594 (85%)
Posterior cerebral artery	51 (67%)	417 (67%)	468 (67%)

eTable 4. Lesion distribution. Values are presented as means and standard deviations [SD] or percentages as appropriate. Note that lesions with at least one voxel overlapping with the mask of an a priori brain region were considered to involve that region.

© 2023 Schaper FLWVJ et al. *JAMA Neurol.*

eFigure 1

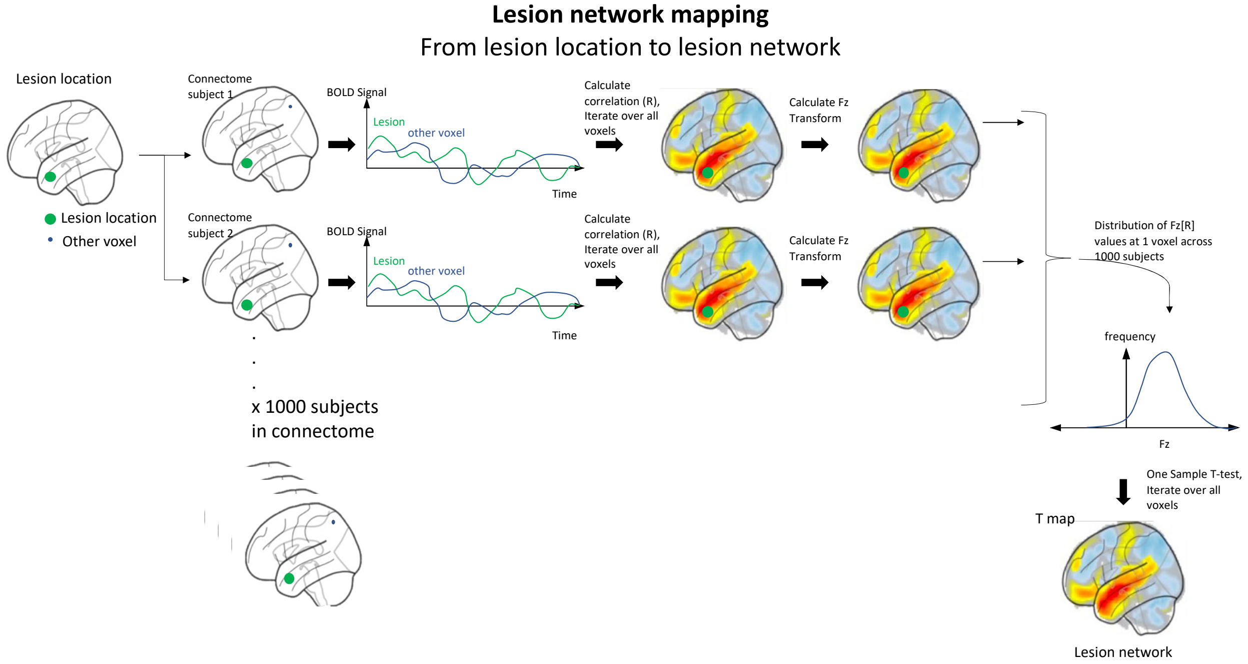

eFigure 1. Lesion network mapping methods flowchart. A lesion network is computed for each lesion location by performing seed-based functional connectivity analyses between the lesion location and all other brain voxels, using the resting state functional connectivity data (2 × 2 × 2 mm resolution) from 1000 healthy participants (human brain connectome: https://dataverse.harvard.edu/dataverse/GSP). First, the correlation (R) between each lesion location's average time course and the time course of every other brain voxel is calculated using the functional connectivity data from each of the 1000 subjects included in the human brain connectome.[19,20] Second, a Fisher's z transformation is applied to each of the 1000 functional connectivity correlation maps to normalize the distribution of values at each voxel. Finally, a T-score is calculated at each voxel (T-map) which represents the statistical relationship of each voxel's functional connectivity to the lesion location, resulting in a lesion network.

© 2023 Schaper FLWVJ et al. *JAMA Neurol.*

eFigure 2

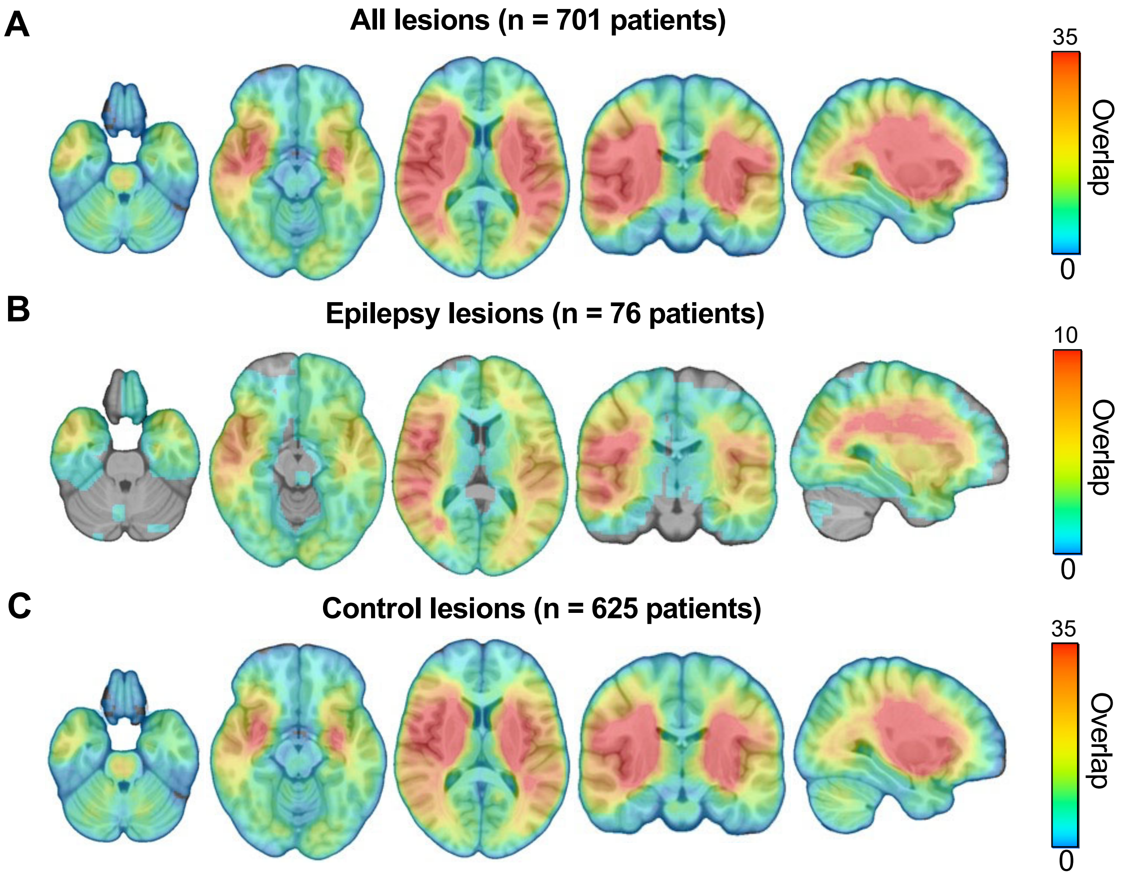

eFigure 2. Lesion overlap. All lesions (**A**), post-stroke epilepsy lesions (**B**), and control stroke lesions (**C**).

© 2023 Schaper FLWVJ et al. *JAMA Neurol.*

eFigure 3

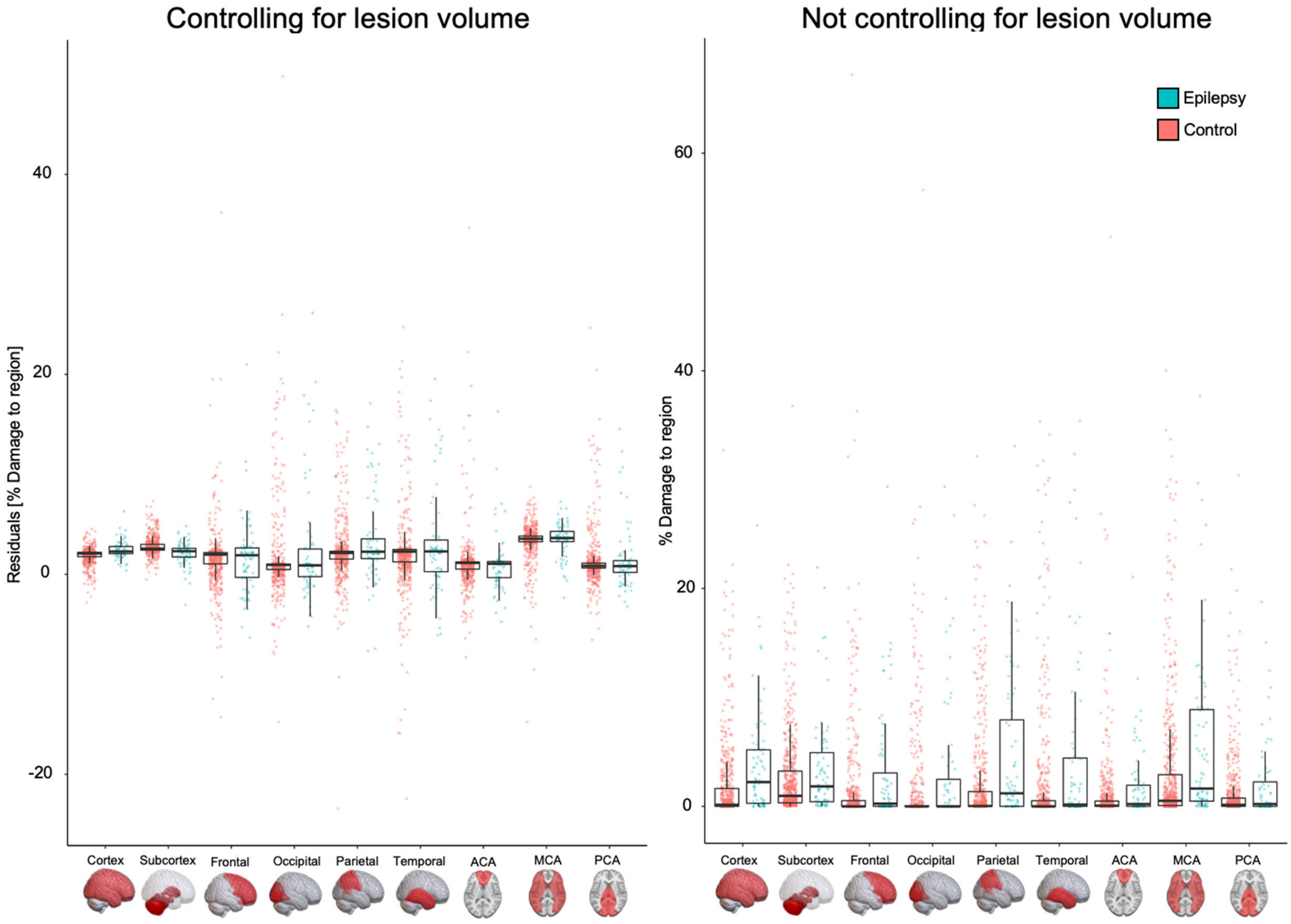

eFigure 3. Distribution of lesion damage to the cortex, subcortex, lobes and vascular territories, with and without correcting for lesion volume.

© 2023 Schaper FLWVJ et al. *JAMA Neurol.*

eFigure 4

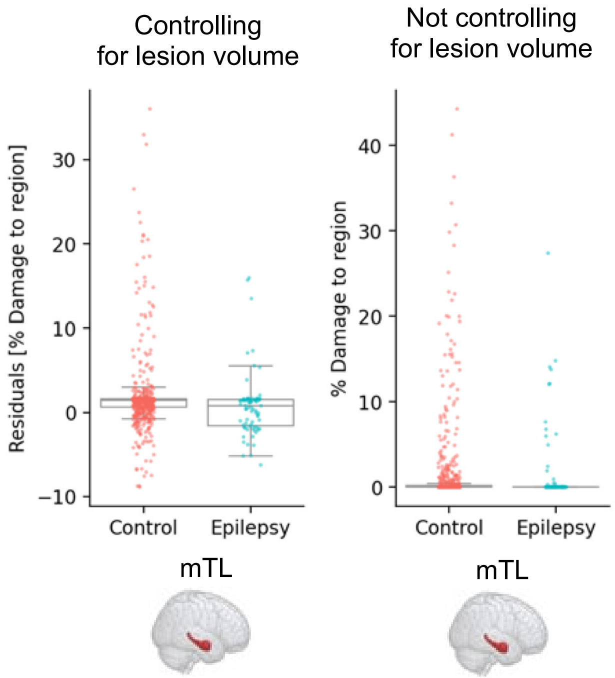

eFigure 4. Distribution of lesion damage to the mesial temporal lobe with and without controlling for lesion volume. *Abbreviations: mTLE, mesial temporal lobe.*

© 2023 Schaper FLWVJ et al. *JAMA Neurol.*

eFigure 5

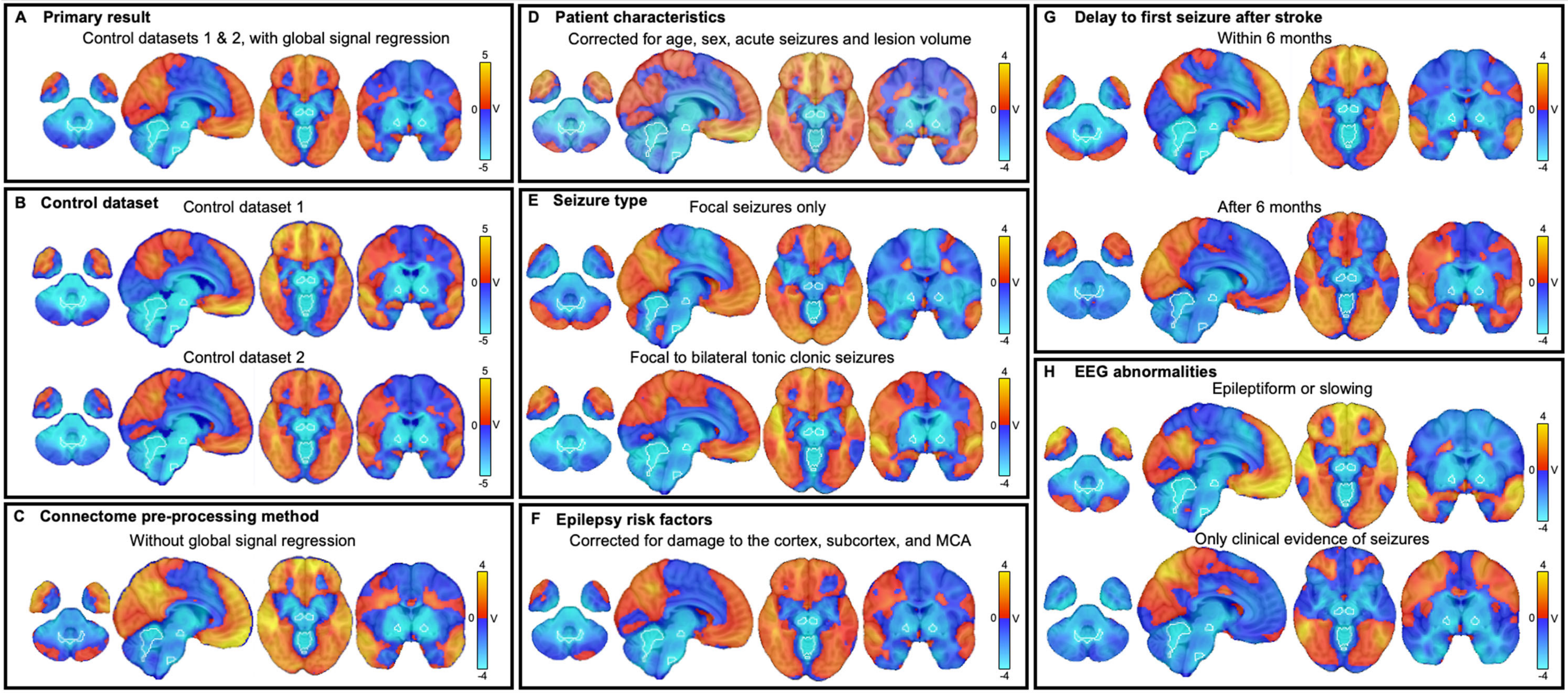

eFigure 5. Lesion network mapping **c**ontrol and subgroup analyses. Our primary lesion network mapping result (**A**) implicating nodes in the cerebellum and basal ganglia (white outline) are similar with different control datasets (**B**), connectome pre-processing methods (**C**), after controlling for patient characteristics (**D**), seizure type (**E**), known epilepsy risk factors (**F**), delay to first seizure after stroke (**G**), and presence of EEG abnormalities or only clinical evidence of seizures (**H**).

© 2023 Schaper FLWVJ et al. *JAMA Neurol.*

eFigure 6

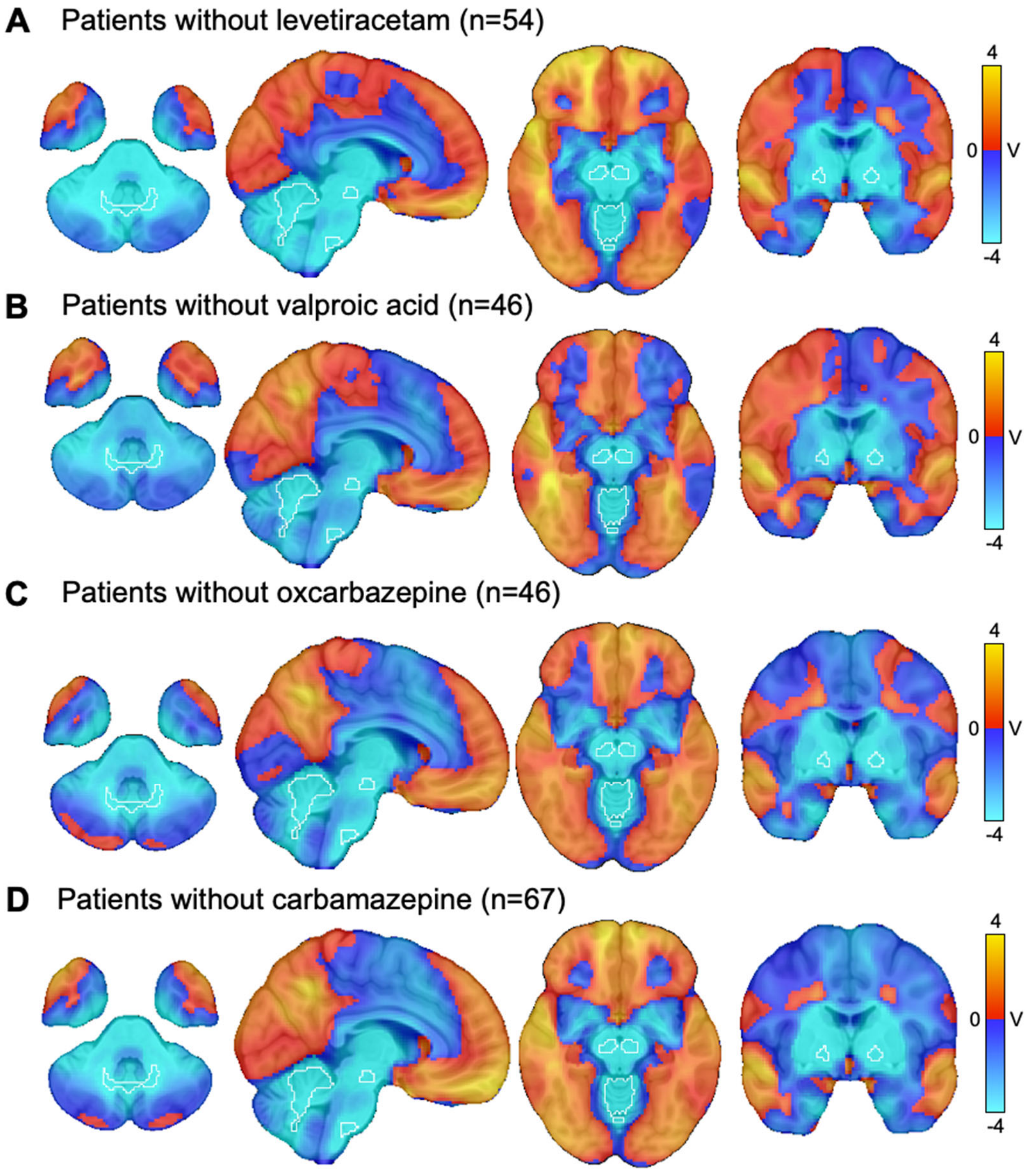

eFigure 6. Lesion network mapping results are similar in subgroups of patients with different antiseizure drugs.

© 2023 Schaper FLWVJ et al. *JAMA Neurol.*

eFigure 7

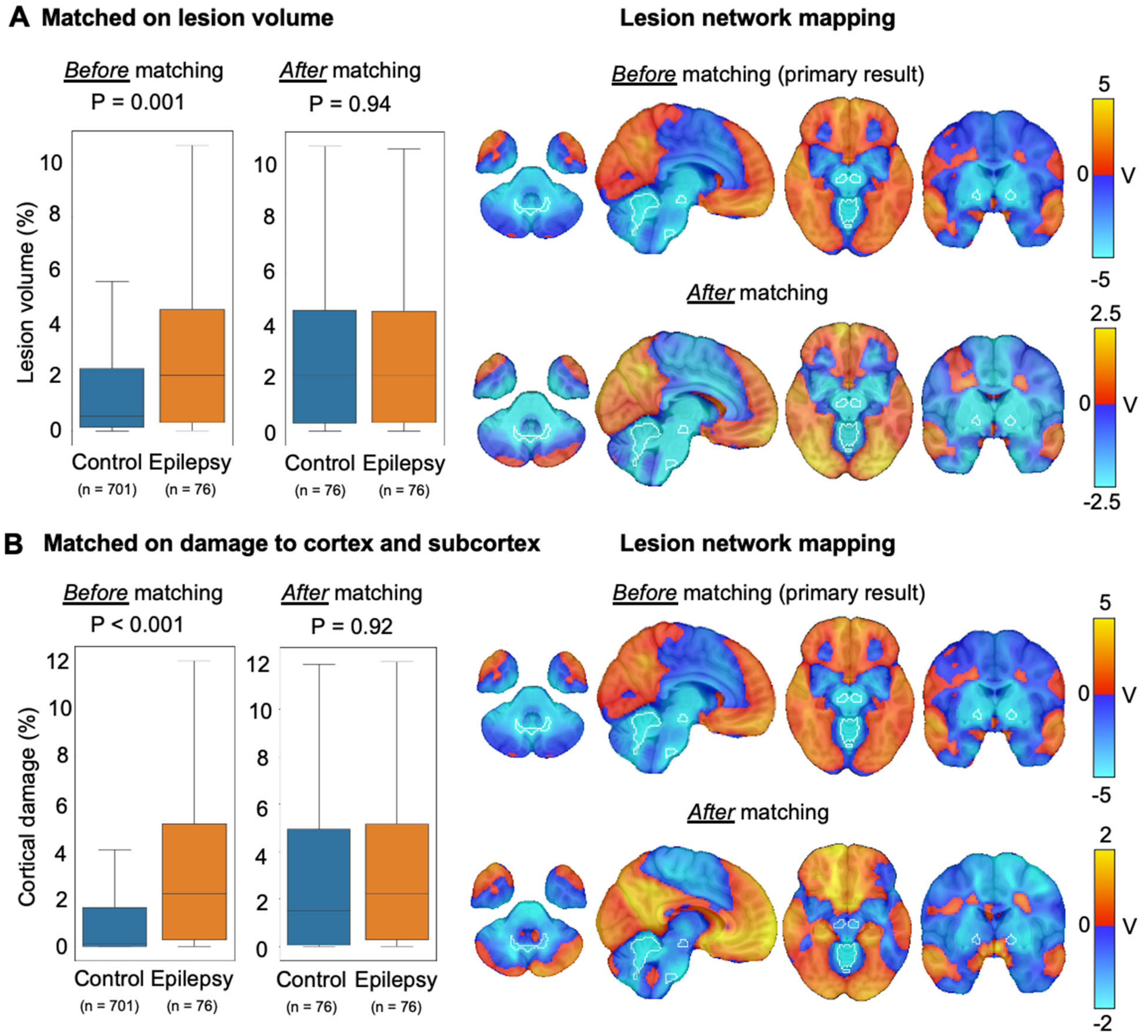

eFigure 7. Lesion network mapping results with matched subgroups. Subgroups were equally matched on lesion volume (**A**) and damage to the cortex and subcortex (**B**). Lesion network mapping results of matched subgroups were consistent with results identified in the total ischemic stroke dataset (white outline). Note that the strength of the relationship (T-values) between connectivity and epilepsy drops consistent with the smaller subgroup (n = 76) compared to our original control (n = 625), but the direction and topography remain the same.

© 2023 Schaper FLWVJ et al. *JAMA Neurol.*

eFigure 8

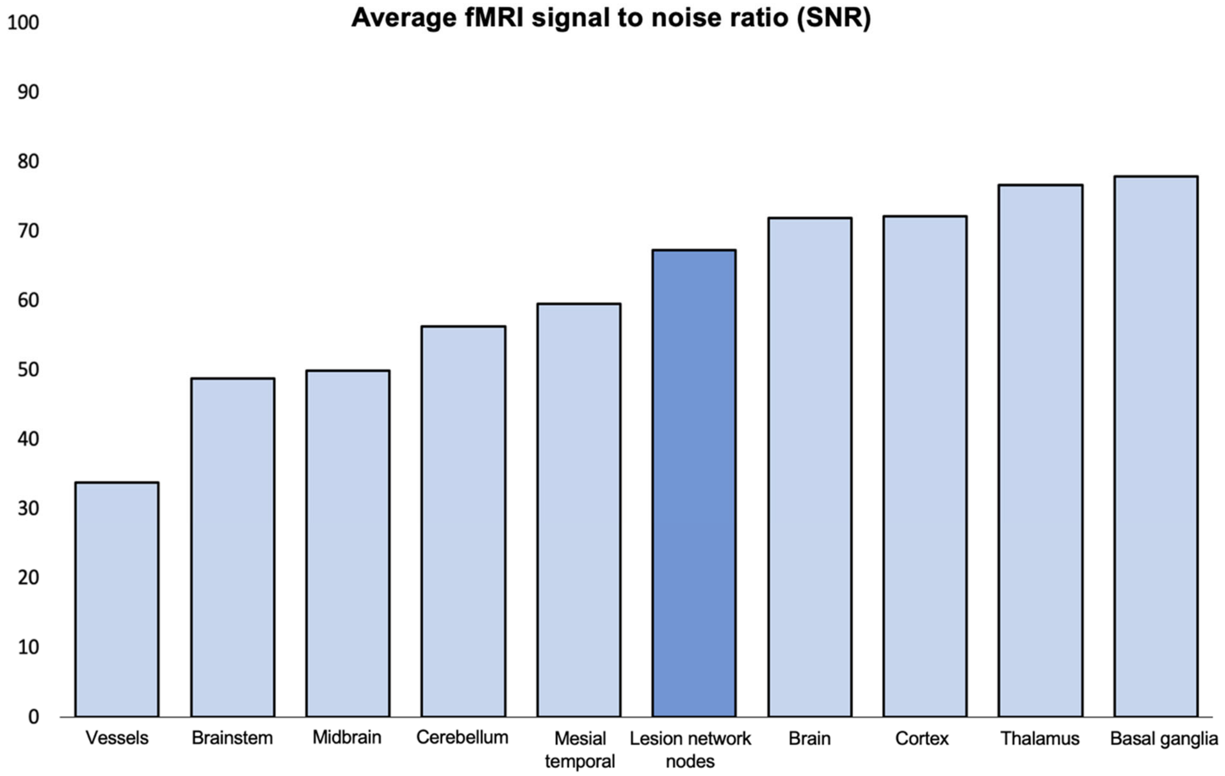

eFigure 8. Temporal signal to noise ratio (SNR) of different brain regions in the functional connectome. Given that our functional connectivity findings mapped to the basal ganglia and cerebellum, we further investigated the temporal SNR within this region of interest (ROI, lesion network nodes in the above figure) to ensure the presence of reliable BOLD signal in this region. We calculated the voxel-wise mean SNR from the Brain Genomics Superstruct Project used in the analyses in the manuscript.[22] fMRI data from each subject was processed through fmriprep, applying standard fMRI preprocessing steps.[51] The SNR (mean of BOLD time series / standard deviation of BOLD time series) was then calculated for each subject using a python script: fmri_SNR.py available here: (https://gist.github.com/alexlicohen/). The group average SNR for the entire brain, the lesion network nodes ROI, and other brain regions were calculated using FSL command line tools.[52] We found that the SNR of the lesion network nodes ROI is similar to the average SNR across the entire brain. Furthermore, the basal ganglia, cerebellum and our specific lesion network nodes within these regions had a SNR > 50 which is considered relatively robust, as prior reports have suggested that SNRs > 20 provide consistent and reproducible functional connectivity results.[53]

eFigure 9

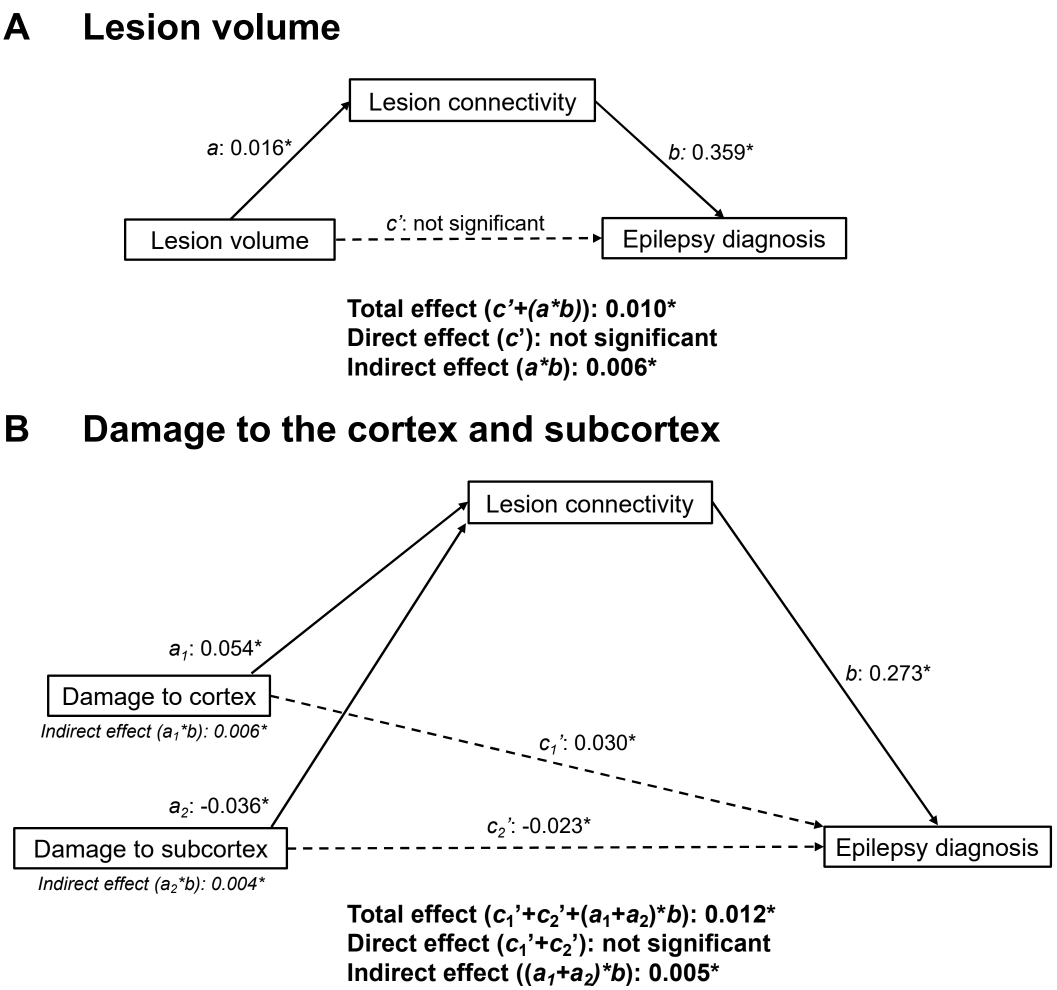

eFigure 9. Statistical mediation analyses. Mediation analysis identified that the relationship between lesion volume and epilepsy diagnosis was fully mediated by lesion connectivity (indirect effect = 0.006, boot standard error [SE] = 0.001, 95% CI = 0.004 to 0.008, **A**). Similarly, the relationship between damage to the cortex and subcortex was also fully mediated by lesion connectivity (indirect effect = 0.005, boot standard error [SE] = 0.001, 95% CI = 0.003 to 0.007, **B**). $^*P < 0.05$

© 2023 Schaper FLWVJ et al. *JAMA Neurol.*

eFigure 10

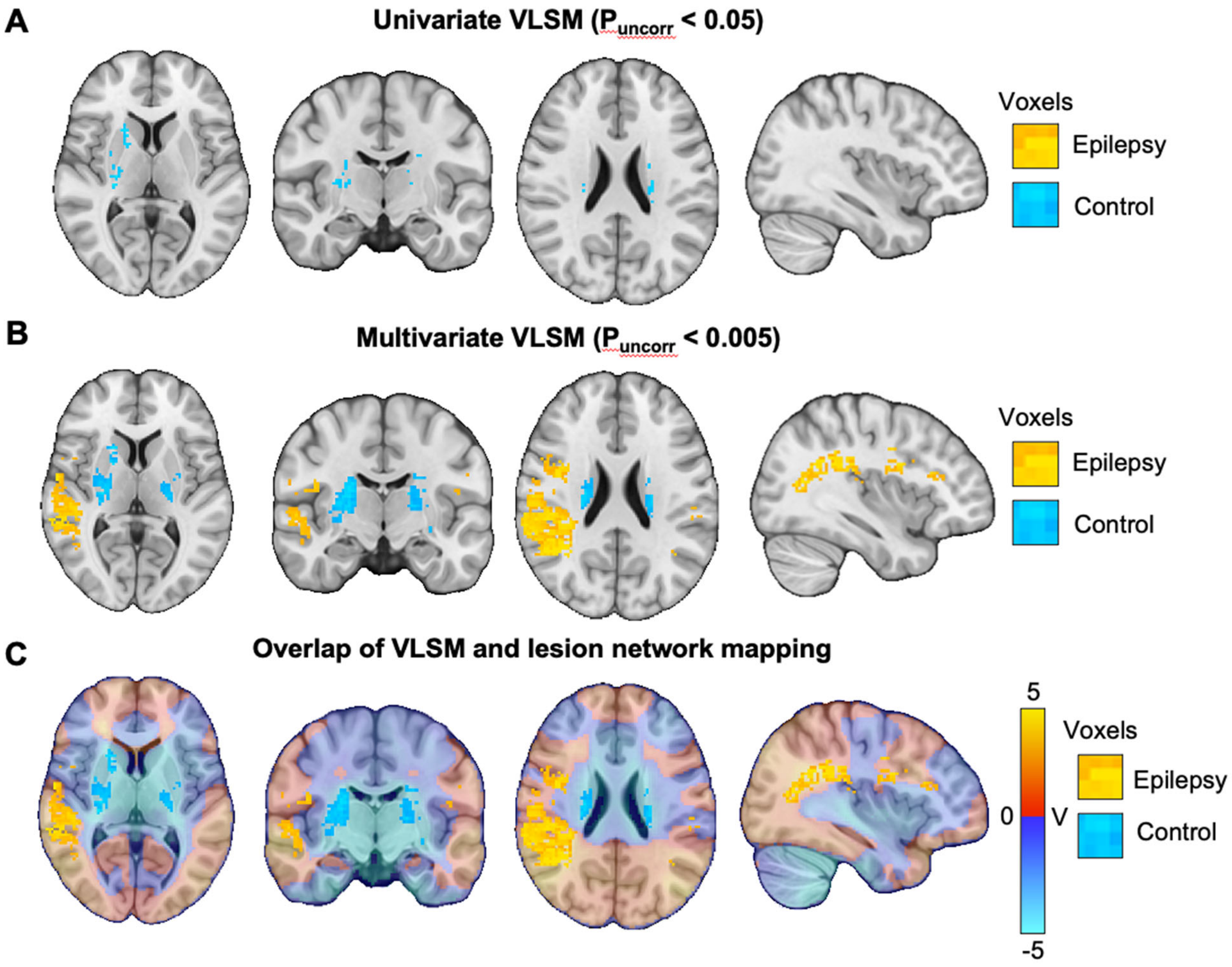

eFigure 10. Voxel-based lesion symptom mapping (VLSM). VLSM results with more liberal statistical cutoffs (P_{uncorr}<0.05) using both univariate (**A**) and multivariate (**B**) methods. VLSM results aligned with lesion network mapping results (**C**) but only identified part of the network.

© 2023 Schaper FLWVJ et al. *JAMA Neurol.*

eFigure 11

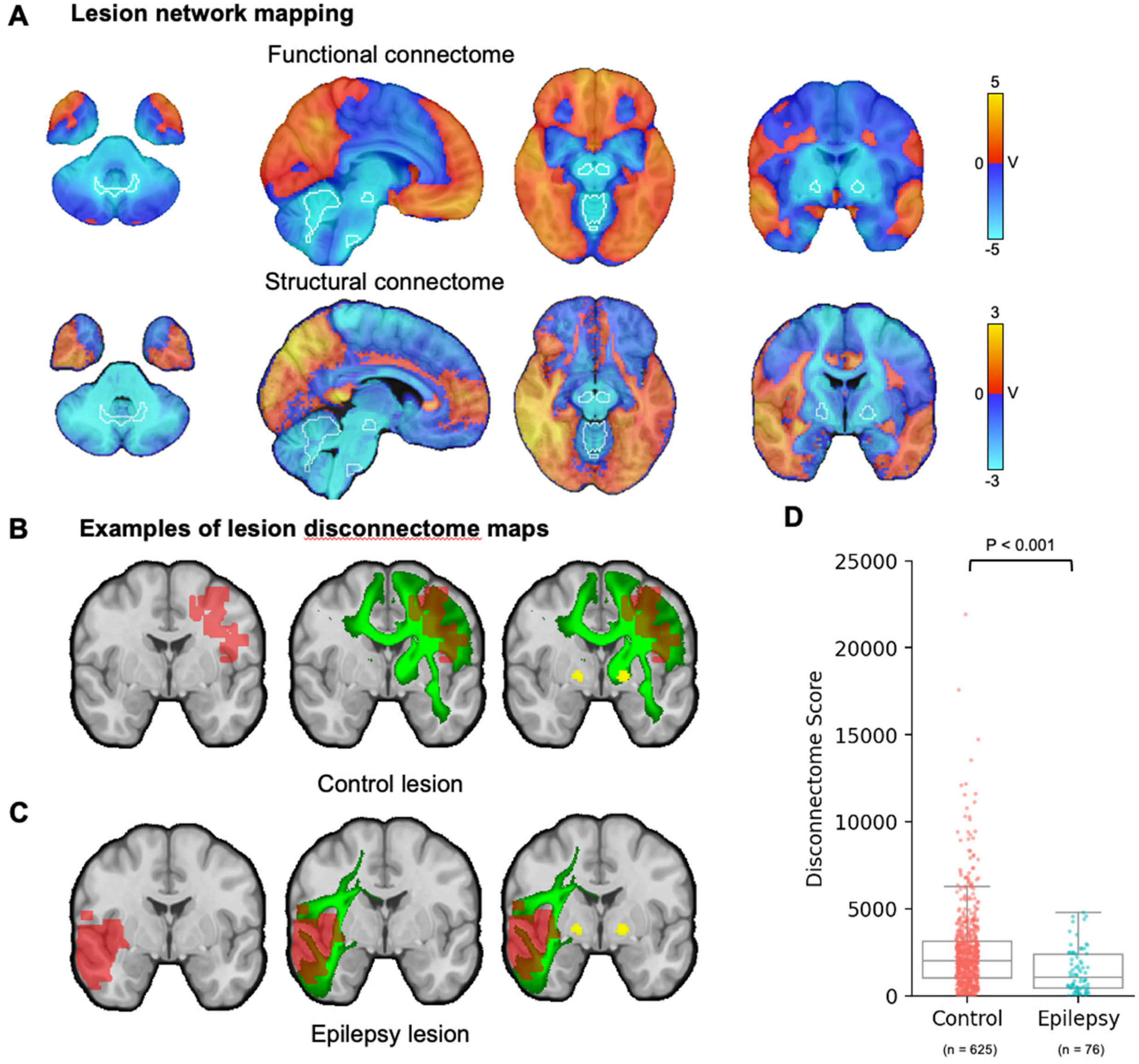

eFigure 11. Lesion network mapping results using a structural connectome. Lesion network mapping results using a structural connectome identified a similar network as using a functional connectome (**A**). In a complimentary analysis, we computed structural disconnection between each lesion location and the lesion network nodes in the basal ganglia and cerebellum (white outlines), and found a significant different between lesions associated with epilepsy (**B**, single example shown) and control lesions (**C**, single example shown). Panels B and C display the lesion location (red, left panel), structural connectivity with the lesion location (green, middle panel), and intersection with our functional lesion network nodes derived using the functional connectome (orange/yellow, right panel). This intersection was used to compute a “disconnectome score” for each lesion, which was significantly different between groups (**D**, Welch’s two-sided test with unequal variances, p < 0.001). Data in panel D are presented as mean (bar) and SD (whiskers) with individual patient values (dots).

eFigure 12

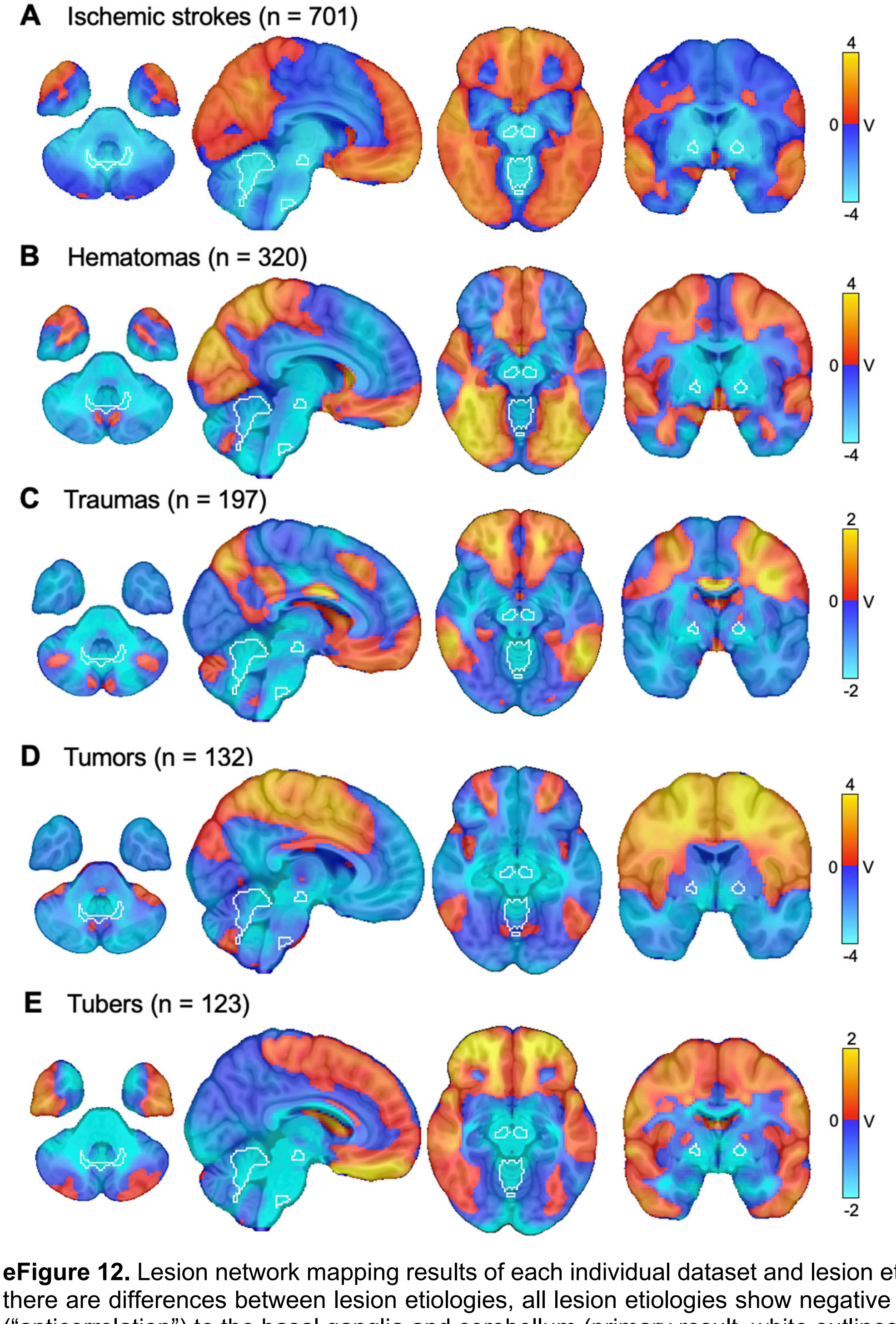

eFigure 12. Lesion network mapping results of each individual dataset and lesion etiology. Note that while there are differences between lesion etiologies, all lesion etiologies show negative functional connectivity (“anticorrelation”) to the basal ganglia and cerebellum (primary result, white outlines).

© 2023 Schaper FLWVJ et al. *JAMA Neurol.*

eFigure 13.

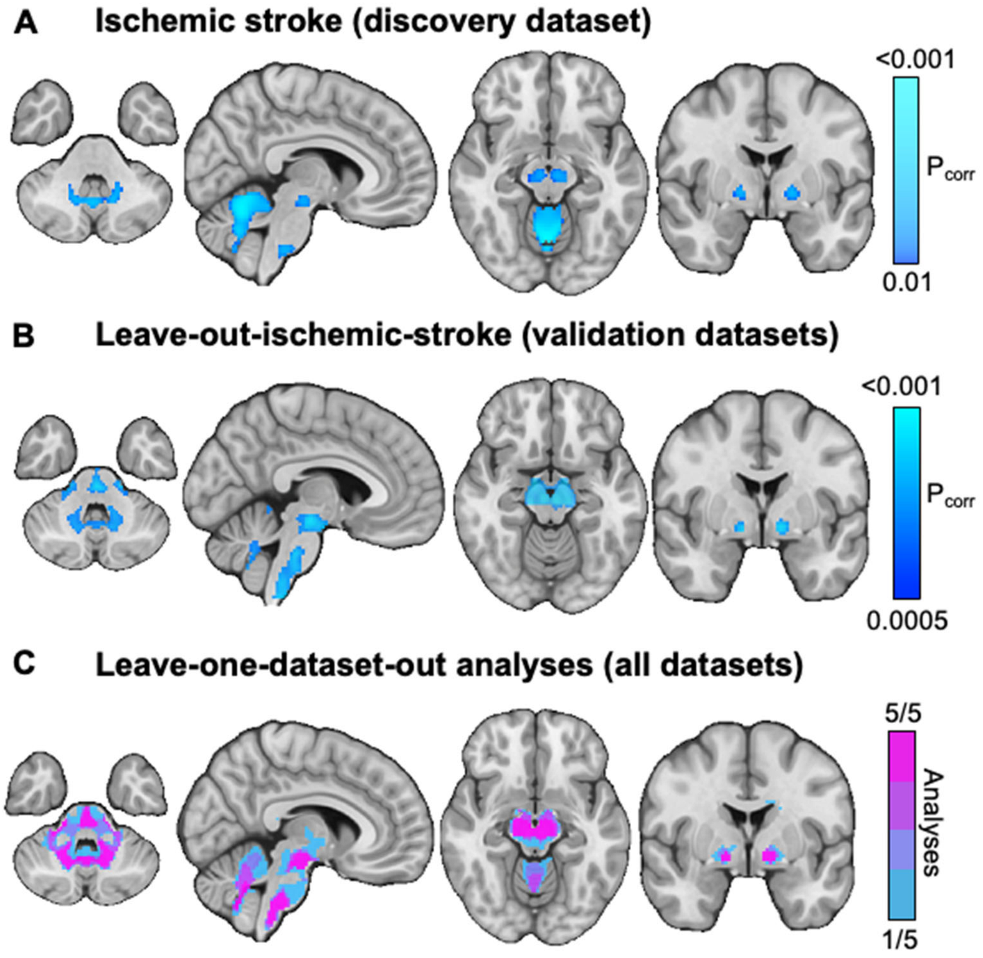

eFigure 13. Whole-brain lesion network mapping results in discovery and validation datasets. To test whether we would identify the same functional connections associated with epilepsy in our validation datasets, as were identified in ischemic stroke (A), we combined the four validation datasets (leaving out ischemic stroke data) and repeated the voxel-based PALM analysis on a whole-brain level, controlling for lesion volume and lesion type. The lesion network nodes significantly associated with epilepsy across these four validation datasets were nearly identical to the initial results from our discovery dataset (B). We then repeated this leave-one-dataset-out analysis five times, each time leaving out a different dataset and identifying functional connections significantly associated with epilepsy across four different lesion types. We identified a high overlap across these 5 different analyses in these same nodes in the basal ganglia and cerebellum (C). P-values are shown after family wise error rate correction for multiple testing.

© 2023 Schaper FLWVJ et al. *JAMA Neurol.*

eFigure 14

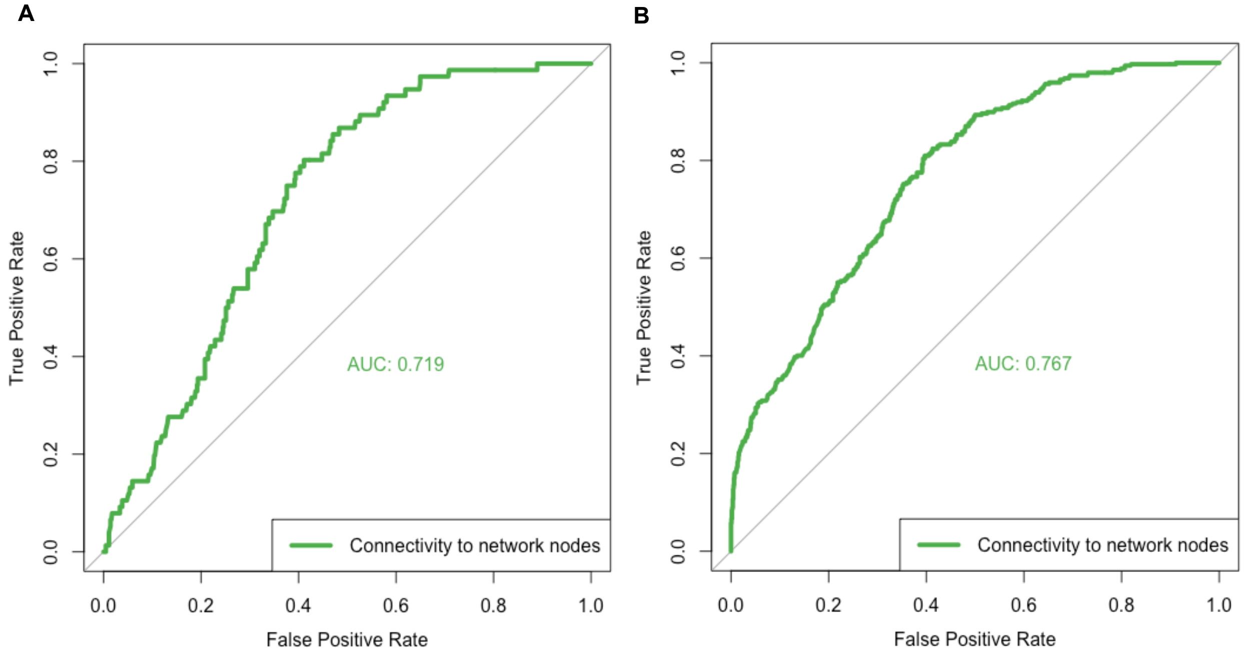

eFigure 14. Receiver operating characteristics (ROC) curves. ROC curves show an association between post-stroke epilepsy (**A**, P<0.001) or across all lesion types (**B**, P<0.001)) and lesion connectivity. To avoid circularity, connectivity was computed between each lesion location and the lesion network nodes derived from the four other lesion type datasets.

eFigure 15

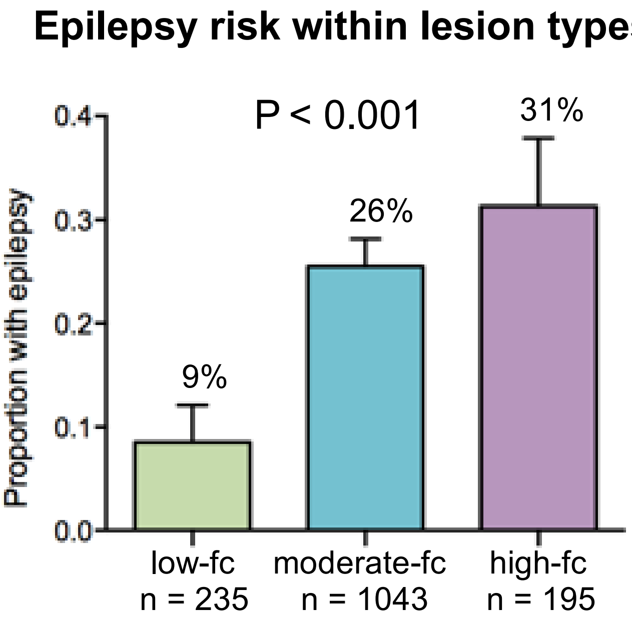

eFigure 15. Proportion of epilepsy in categories based on lesion connectivity. Lesion connectivity categories are associated with the proportion of epilepsy ($\chi^2 = 38.2$, df = 2, $P < 0.001$). Connectivity was computed from each lesion location to the nodes derived from the other four lesion type datasets (leave-one-lesion-type-out). Note that this analysis is similar to the analysis presented in main text (see Figure 4B), but risk was assessed within each lesion type rather than across all lesion types.

© 2023 Schaper FLWVJ et al. *JAMA Neurol.*

eFigure 16

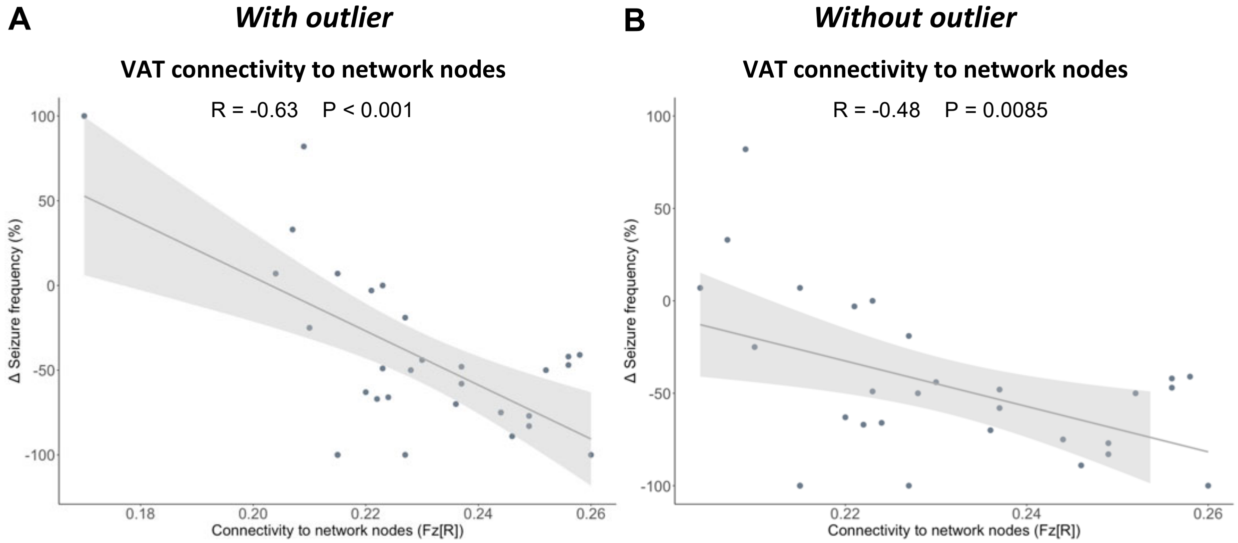

eFigure 16. Correlation between DBS site connectivity and clinical outcome. DBS site connectivity to the lesion network nodes derived from lesion network mapping correlated with a reduction in seizure frequency after DBS. Correlation was computed with (A) and without (B) an outlier that had worsened seizure control after DBS. DBS parameters were not significantly correlated with seizure frequency, including amplitude (r = -0.22, P = 0.24), VAT volume (r = -0.31, P = 0.11), frequency (r = 0.001, P = 0.99) or pulse width (r = 0.1, P = 0.62).

© 2023 Schaper FLWVJ et al. *JAMA Neurol.*

eFigure 17

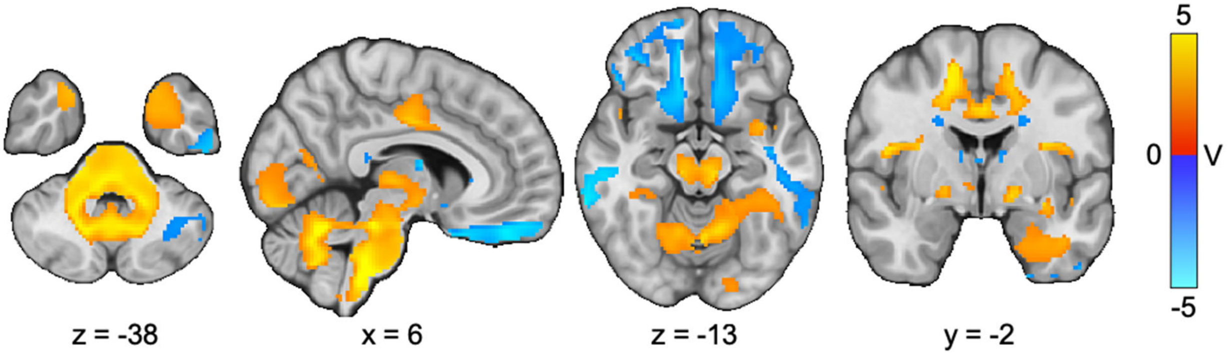

eFigure 17. Whole-brain DBS network mapping results. DBS network mapping on a whole-brain level implicates the same nodes in the cerebellum and basal ganglia (P_{corr}<0.05) as shown within the restricted ROI (see main manuscript text and Figure 5D). Results are shown after family-wise error correction for multiple testing and threshold free cluster enhancement.

© 2023 Schaper FLWVJ et al. *JAMA Neurol.*

References

1. Nordberg J, Schaper FL, Bucci M, Nummenmaa L, Joutsa J. Brain lesion locations associated with secondary seizure generalization. *medRxiv*. Published online January 2021:2021.04.19.21255414.

2. Jenkinson M, Smith S. A global optimisation method for robust affine registration of brain images. *Medical Image Analysis*. 2001;5(2):143-156.

3. Sperber C, Karnath HO. On the validity of lesion-behaviour mapping methods. *Neuropsychologia*. 2018;115:17-24.

4. Pustina D, Doucet G, Evans J, et al. Distinct Types of White Matter Changes Are Observed after Anterior Temporal Lobectomy in Epilepsy. *PloS one*. 2014;9(8):e104211-13.

5. Corbetta M, Ramsey L, Callejas A, et al. Common behavioral clusters and subcortical anatomy in stroke. *Neuron*. 2015;85(5):927-941.

6. Wu O, Cloonan L, Mocking SJT, et al. Role of Acute Lesion Topography in Initial Ischemic Stroke Severity and Long-Term Functional Outcomes. *Stroke*. 2015;46(9):2438-2444.

7. Galovic M, Döhler N, Erdélyi-Canavese B, et al. Prediction of late seizures after ischaemic stroke with a novel prognostic model (the SeLECT score): a multivariable prediction model development and validation study. *Lancet neurology*. 2018;17(2):143-152.

8. Desikan RS, Ségonne F, Fischl B, et al. An automated labeling system for subdividing the human cerebral cortex on MRI scans into gyral based regions of interest. *NeuroImage*. 2006;31(3):968-980.

9. Collins DL, Holmes CJ, Peters TM, Evans AC. Automatic 3-D model-based neuroanatomical segmentation. *Human Brain Mapping*. 1995;3(3):190-208.

10. Schirmer MD, Giese AK, Fotiadis P, et al. Spatial Signature of White Matter Hyperintensities in Stroke Patients. *Frontiers in neurology*. 2019;10:208.

11. Winkler AM, Ridgway GR, Webster MA, Smith SM, Nichols TE. Permutation inference for the general linear model. *NeuroImage*. 2014;92:381-397.

12. Sperber C, Karnath HO. Impact of correction factors in human brain lesion-behavior inference. *Human Brain Mapping*. 2017;38(3):1692-1701.

13. Stark. *User Manual and Tutorial for NiiStat*.; 2018.

14. Mah YH, Husain M, Rees G, Nachev P. Human brain lesion-deficit inference remapped. *Brain*. 2014;137(9):2522-2531.

15. Zhang Y, Kimberg DY, Coslett HB, Schwartz MF, Wang Z. Multivariate lesion-symptom mapping using support vector regression. *Hum Brain Mapp*. 2014;35(12):5861-5876.

© 2023 Schaper FLWVJ et al. *JAMA Neurol.*

16. DeMarco AT, Turkeltaub PE. A multivariate lesion symptom mapping toolbox and examination of lesion-volume biases and correction methods in lesion-symptom mapping. *Human Brain Mapping*. 2018;39(11):4169-4182.

17. Karnath HO, Sperber C, Rorden C. Mapping human brain lesions and their functional consequences. *NeuroImage*. 2018;165:180-189.

18. Fox MD. Mapping Symptoms to Brain Networks with the Human Connectome. *The New England journal of medicine*. 2018;379(23):2237-2245.

19. Boes AD, Prasad S, Liu H, et al. Network localization of neurological symptoms from focal brain lesions. *Brain : a journal of neurology*. 2015;138(Pt 10):3061-3075.

20. Darby RR, Laganiere S, Pascual-Leone A, Prasad S, Fox MD. Finding the imposter: brain connectivity of lesions causing delusional misidentifications. *Brain : a journal of neurology*. 2017;140(2):497-507.

21. Yeo BTT, Krienen FM, Sepulcre J, et al. The organization of the human cerebral cortex estimated by intrinsic functional connectivity. *Journal of neurophysiology*. 2011;106(3):1125-1165.

22. Holmes AJ, Hollinshead MO, O'Keefe TM, et al. Brain Genomics Superstruct Project initial data release with structural, functional, and behavioral measures. *Scientific data*. 2015;2:150031.

23. Cotovio G, Talmasov D, Barahona-Corrêa JB, et al. Mapping mania symptoms based on focal brain damage. *Journal of Clinical Investigation*. 2020;130(10):5209-5222.

24. Padmanabhan JL, Cooke D, Joutsa J, et al. A Human Depression Circuit Derived From Focal Brain Lesions. *Biological psychiatry*. 2019;86(10):749-758.

25. Fox MD, Snyder AZ, Vincent JL, Corbetta M, Van Essen DC, Raichle ME. The human brain is intrinsically organized into dynamic, anticorrelated functional networks. *Proceedings of the National Academy of Sciences of the United States of America*. 2005;102(27):9673-9678.

26. Murphy K, Fox MD. Towards a consensus regarding global signal regression for resting state functional connectivity MRI. *Neuroimage*. 2017;154:169-173.

27. Li J, Kong R, Liégeois R, et al. Global signal regression strengthens association between resting-state functional connectivity and behavior. *NeuroImage*. Published online April 2019.

28. Cohen AL, Soussand L, Corrow SL, Martinaud O, Barton JJS, Fox MD. Looking beyond the face area: lesion network mapping of prosopagnosia. *Brain : a journal of neurology*. 2019;142(12):3975-3990.

29. Behzadi Y, Restom K, Liau J, Liu TT. A component based noise correction method (CompCor) for BOLD and perfusion based fMRI. *NeuroImage*. 2007;37(1):90-101.

© 2023 Schaper FLWVJ et al. *JAMA Neurol.*

30. Whitfield-Gabrieli S, Nieto-Castanon A. Conn: A Functional Connectivity Toolbox for Correlated and Anticorrelated Brain Networks. *Brain Connectivity*. 2012;2(3):125-141.

31. Rubin D, Rosenbaum P. Constructing a Control Group Using Multivariate Matched Sampling Methods That Incorporate the Propensity Score. *The American Statistician*. 1985;39.

32. D'Agostino R. Propensity score methods for bias reduction in the comparison of a treatment to a non-randomized control group. *Stat Med*. 1998;17(19):2265-2281.

33. Ho D, Imai K, King G, Stuart EA. MatchIt: Nonparametric Preprocessing for Parametric Causal Inference. *Journal of Statistical Software*. 2011;42:1-28.

34. Staffa SJ, Zurakowski D. Five Steps to Successfully Implement and Evaluate Propensity Score Matching in Clinical Research Studies. *Anesthesia & Analgesia*. 2018;127(4):1066-1073.

35. Rosseel Y. lavaan: An R Package for Structural Equation Modeling. *Journal of Statistical Software*. 2012;48:1-36. doi:10.18637/jss.v048.i02

36. Joutsa J, Moussawi K, Siddiqi SH, et al. Brain lesions disrupting addiction map to a common human brain circuit. *Nat Med*. 2022;28(6):1249-1255.

37. de Greef BTA, Schreuder FHBM, Vlooswijk MCG, et al. Early seizures after intracerebral hemorrhage predict drug-resistant epilepsy. *J Neurol*. 2015;262(3):541-546.

38. Raymont V, Salazar AM, Lipsky R, Goldman D, Tasick G, Grafman J. Correlates of posttraumatic epilepsy 35 years following combat brain injury. *Neurology*. 2010;75(3):224-229.

39. Cayuela N, Simó M, Majós C, et al. Seizure-susceptible brain regions in glioblastoma: identification of patients at risk. *European journal of neurology : the official journal of the European Federation of Neurological Societies*. 2018;25(2):387-394.

40. Cohen AL, Mulder BPF, Prohl AK, et al. Tuber Locations Associated with Infantile Spasms Map to a Common Brain Network. *Annals of neurology*. 2021;12:2825.

41. Sharrock MF, Mould WA, Ali H, et al. 3D Deep Neural Network Segmentation of Intracerebral Hemorrhage: Development and Validation for Clinical Trials. *Neuroinformatics*. 2021;19(3):403-415.

42. Winkler AM, Webster MA, Vidaurre D, Nichols TE, Smith SM. Multi-level block permutation. *NeuroImage*. 2015;123:253-268.

43. Faul F, Erdfelder E, Lang AG, Buchner A. G*Power 3: a flexible statistical power analysis program for the social, behavioral, and biomedical sciences. *Behav Res Methods*. 2007;39(2):175-191.

© 2023 Schaper FLWVJ et al. *JAMA Neurol.*

44. Schaper FLWVJ, Plantinga BR, Colon AJ, et al. Deep Brain Stimulation in Epilepsy: A Role for Modulation of the Mammillothalamic Tract in Seizure Control? *Neurosurgery*. 2020;51(5):899.

45. Horn A, Kühn AA. Lead-DBS: a toolbox for deep brain stimulation electrode localizations and visualizations. *NeuroImage*. 2015;107:127-135.

46. Horn A, Reich M, Vorwerk J, et al. Connectivity predicts deep brain stimulation outcome in Parkinson's disease. *Ann Neurol*. 2017;82:67-78.

47. Friston KJ, Holmes AP, Worsley KJ, Poline JP, Frith CD, Frackowiak RSJ. Statistical parametric maps in functional imaging: A general linear approach. *Human Brain Mapping*. 1994;2(4):189-210.

48. Schönecker T, Kupsch A, Kühn AA, Schneider GH, Hoffmann KT. Automated Optimization of Subcortical Cerebral MR Imaging−Atlas Coregistration for Improved Postoperative Electrode Localization in Deep Brain Stimulation. *Am J Neuroradiol*. 2009;30(10):1914.

49. Avants BB, Epstein CL, Grossman M, Gee JC. Symmetric diffeomorphic image registration with cross-correlation: Evaluating automated labeling of elderly and neurodegenerative brain. *Medical Image Analysis*. 2008;12(1):26-41.

50. Vorwerk J, Oostenveld R, Piastra MC, Magyari L, Wolters CH. The FieldTrip-SimBio pipeline for EEG forward solutions. *BioMedical Engineering OnLine*. 2018;17(1):37.

51. Esteban O, Markiewicz CJ, Blair RW, et al. fMRIPrep: a robust preprocessing pipeline for functional MRI. *Nat Methods*. 2019;16(1):111-116.

52. Jenkinson M, Beckmann CF, Behrens TEJ, Woolrich MW, Smith SM. FSL. *NeuroImage*. 2012;62(2):782-790.

53. Barry RL, Conrad BN, Smith SA, Gore JC. A practical protocol for measurements of spinal cord functional connectivity. *Sci Rep*. 2018;8(1):16512.

© 2023 Schaper FLWVJ et al. *JAMA Neurol.*
