# Supplementary material for: Mapping Lesion-Related Epilepsy to a Human Brain Network
Source: JAMA Neurol. 2023 Jul 3;80(9):891–902. doi: 10.1001/jamaneurol.2023.1988 (PMC10318550; doi:10.1001/jamaneurol.2023.1988)
Supplement: Supplement 2. — Data sharing statement [file jamaneurol-e231988-s002.pdf]

Data Sharing Statement

Data
Data available: No

Additional Information
Explanation for why data not available: This paper used de-identified data from eight different teams of investigators at various institutions, across different countries. Each dataset is available upon reasonable request from each respective team of investigators. Data sharing will be subject to the policies and procedures of the institution as well as the laws of the country where each dataset was collected.
